# DNAm landscape up to 4 months post SARS-CoV-2 infection: insights from four population-based cohorts

**DOI:** 10.1186/s13148-026-02166-1

**Published:** 2026-06-13

**Authors:** Pamela R. Matías-García, Liye Lai, Thomas Delerue, Jochen Ohnmacht, Klaus J. Stark, Robert Warmerdam, Alexey Kolodkin, Julia Six-Merker, Nina Mangold, Kathrin Günther, Marc P. O’Sullivan, Armin Rauschenberger, Barbara Bohn, Klaus Berger, Julia Fricke, Peter Ahnert, Lude Franke, Rejko Krüger, Olaf Gefeller, Klaus Überla, Iris M. Heid, Ralf Wagner, Lifelines Corona Research Initiative, TiKoCo Working Group, CON-VINCE Consortium, NAKO Investigators, Melanie Waldenberger, Annette Peters

**Affiliations:** 1 Research Unit Molecular Epidemiology, Helmholtz Munich, Neuherberg, Germany; 2 Institute of Epidemiology, Helmholtz Munich, Neuherberg, Germany; 3 German Research Center for Cardiovascular Disease (DZHK), partner site Munich Heart Alliance, Munich, Germany; 4 Institute for Medical Information Processing, Biometry, and Epidemiology (IBE), Faculty of Medicine, Ludwig-Maximilians-Universität München, Munich, Germany; 5 Luxembourg Institute of Health, Strassen, Luxembourg; 6 Department of Genetic Epidemiology, University of Regensburg, Regensburg, Germany; 7 Department of Genetics, University of Groningen, University Medical Center Groningen, Groningen, The Netherlands; 8 Oncode Institute, Utrecht, The Netherlands; 9 Department for Diagnostic und Interventional Radiology, University Hospital Augsburg, Augsburg, Germany; 10 Leibniz Institute for Prevention Research and Epidemiology - BIPS, Bremen, Germany; 11 Luxembourg Centre for Systems Biomedicine (LCSB), University of Luxembourg, Esch-sur-Alzette, Luxembourg; 12 NAKO e.V., Heidelberg, Germany; 13 Institute of Epidemiology and Social Medicine, University of Münster, Münster, Germany; 14 Institute of Social Medicine, Epidemiology and Health Economics, Charité – Universitätsmedizin Berlin, Berlin, Germany; 15 Unit for Municipal Health Strategies for the City of Freiburg and the District of Breisgau-Hochschwarzwald, Freiburg, Germany; 16 Institute for Medical Informatics, Statistics and Epidemiology, University of Leipzig, Leipzig, Germany; 17 Centre Hospitalier du Luxembourg, Strassen, Luxembourg; 18 Department of Medical Informatics, Biometry and Epidemiology, Friedrich- Alexander-Universität Erlangen-Nürnberg (FAU), Erlangen, Germany; 19 Institute of Clinical and Molecular Virology, University Hospital Erlangen, Friedrich-Alexander-Universität Erlangen-Nürnberg, Erlangen, Germany; 20 Institute of Medical Microbiology and Hygiene, Molecular Microbiology (Virology), University of Regensburg, Regensburg, Germany; 21 Institute of Clinical Microbiology and Hygiene, University Hospital Regensburg, Regensburg, Germany

**Keywords:** COVID-19, SARS-CoV-2, EWAS, DNA methylation, ORCHESTRA cohort, Epigenetic changes, Population-based

## Abstract

**Background:**

Evidence for persistent epigenetic changes in individuals who had a mild SARS-CoV-2 infection is limited, as most DNA methylation (DNAm) studies to date have focused on either the acute phase of infection or on the months following infection in severe cases requiring hospitalization.

**Methods and results:**

Using the Infinium Human MethylationEPIC BeadChip, we investigated blood DNA methylation (DNAm) up to four months after SARS-CoV-2 infection in cases and controls from four population-based cohorts (NAKO, Lifelines, CON-VINCE, and TiKoCo; *n* = 675) within the framework of the ORCHESTRA Consortium. We observed DNAm changes at 16 differentially methylated positions (DMPs) and 21 differentially methylated regions (DMRs), with 89% of these DMPs/DMRs hypomethylated in cases compared to age- and sex-matched controls. Genes mapped to these CpGs were annotated with Gene Ontology terms and pathways related to immune responses to viral infection. eQTM analyses in whole blood from an independent cohort (KORA FF4 study) produced 49 significant CpG–transcript pairs, including *IFI44L* and *GNA12*. Despite inter-individual variability and cohort heterogeneity, our findings regarding four DMPs (*IFI44L*, *MX1*, *DDX60*, and *RABGAP1L*) and two DMRs (*PARP9* and *GNA12*) replicate changes described both in the acute phase of infection and at long-term follow-up. Differential methylation at other novel loci may reflect the systemic nature of post-infection epigenetic changes.

**Conclusion:**

Our findings suggest moderate but persistent epigenetic changes up to four months after SARS-CoV-2 infection in mild cases from population-based cohorts. These changes partially mirror those reported during the acute phase of both mild and severe COVID-19 and overlap with pathways dysregulated in autoimmune, metabolic and neurological disease. Future research should examine epigenetic changes associated with persisting symptoms in long COVID, investigate downstream effects of DNAm changes on other -omics, and consider longer follow-up periods to further elucidate the molecular mechanisms underlying SARS-CoV-2 induced epigenetic changes.

**Supplementary Information:**

The online version contains supplementary material available at 10.1186/s13148-026-02166-1.

## Introduction

Changes in DNA methylation (DNAm) levels have been widely investigated in epigenome-wide association studies (EWAS) during the acute phase of SARS-CoV-2 infection, particularly in critically ill patients requiring hospitalization. Studies have described DNAm signatures correlating with disease progression or distinguishing between cases and controls [1–7]. These studies have demonstrated significant shifts in white blood cell composition and associated epigenetic and transcriptomic changes that drive COVID-19 disease progression [1, 2, 8]. Some epigenome-wide studies have focused on identifying DNAm changes predictive of COVID-19 outcomes. Castro de Moura et al. identified a 44-CpG epigenetic signature predictive of disease severity in mild and severe COVID-19 patients without comorbidities [3]. Balnis et al. identified a COVID-19-specific signature with 77 differentially methylated regions (DMR) predictive of disease severity in hospitalized patients [4]. Konigsberg and colleagues reported 13,033 CpGs associated with case status and DNAm signatures linked to disease progression and hospitalization [5]. Similarly, Calzari et al. identified DNAm markers predictive of severe infection in comparison to mild disease in individuals with comorbidities [7]. Additionally, several studies have sought to distinguish COVID-19 specific DNAm signatures by comparing them with DNAm profiles from individuals with other respiratory and infectious diseases [4, 6]. Despite differences in study designs – such as sample sizes, patient populations, and analytical methods –, common findings across these studies indicate that widespread hypomethylation in severe cases [1, 2], as well as changes in genes and pathways related to interferon response to viral infection [1–3, 5, 6].

In contrast, considerably fewer studies have investigated epigenetic changes associated with milder forms of COVID-19 [8, 9] and/or with longer follow-up periods post-infection [9–13]. A longitudinal study examining asymptomatic and mildly symptomatic infection in 133 young adults found that differentially methylated sites persisted for several months [8]. The study with the longest follow-up assessed epigenetic changes in 15 patients one year after hospital discharge identifying 71 persistently dysregulated differentially methylated regions (DMRs) out of 1,505 DMRs associated with acute illness at baseline [4, 10]. Two additional EWAS have identified changes three to six months post-infection [11, 13]: one study, analysing blood samples collected 8–12 weeks after SARS-CoV-2 infection in 109 patients and 73 controls, identified three hypomethylated CpGs in cases [11]. Another, examining 96 post-COVID individuals six months after infection alongside 191 pre-pandemic controls, found 42 differentially methylated CpGs [13].

These studies provide insights into the long-term epigenetic changes associated with severe COVID-19 in comparison to mild disease or pre-pandemic controls. However, most lack the statistical power to detect smaller effects linked to mild disease. Consequently, evidence on the epigenetic signature of mild COVID-19 remains scarce, particularly when compared to exposed controls. Furthermore, it remains unclear how these changes correlate with those observed during the acute phase of infection and whether they persist for months post-infection. This study aims to thus identify differentially methylated CpGs in individuals from population-based cohorts in the largest EWAS to date in order to characterize the epigenetic landscape up to four months following mild SARS-CoV-2 infection.

## Methods

### Cohort descriptions and participant selection

**Fig. 1:**
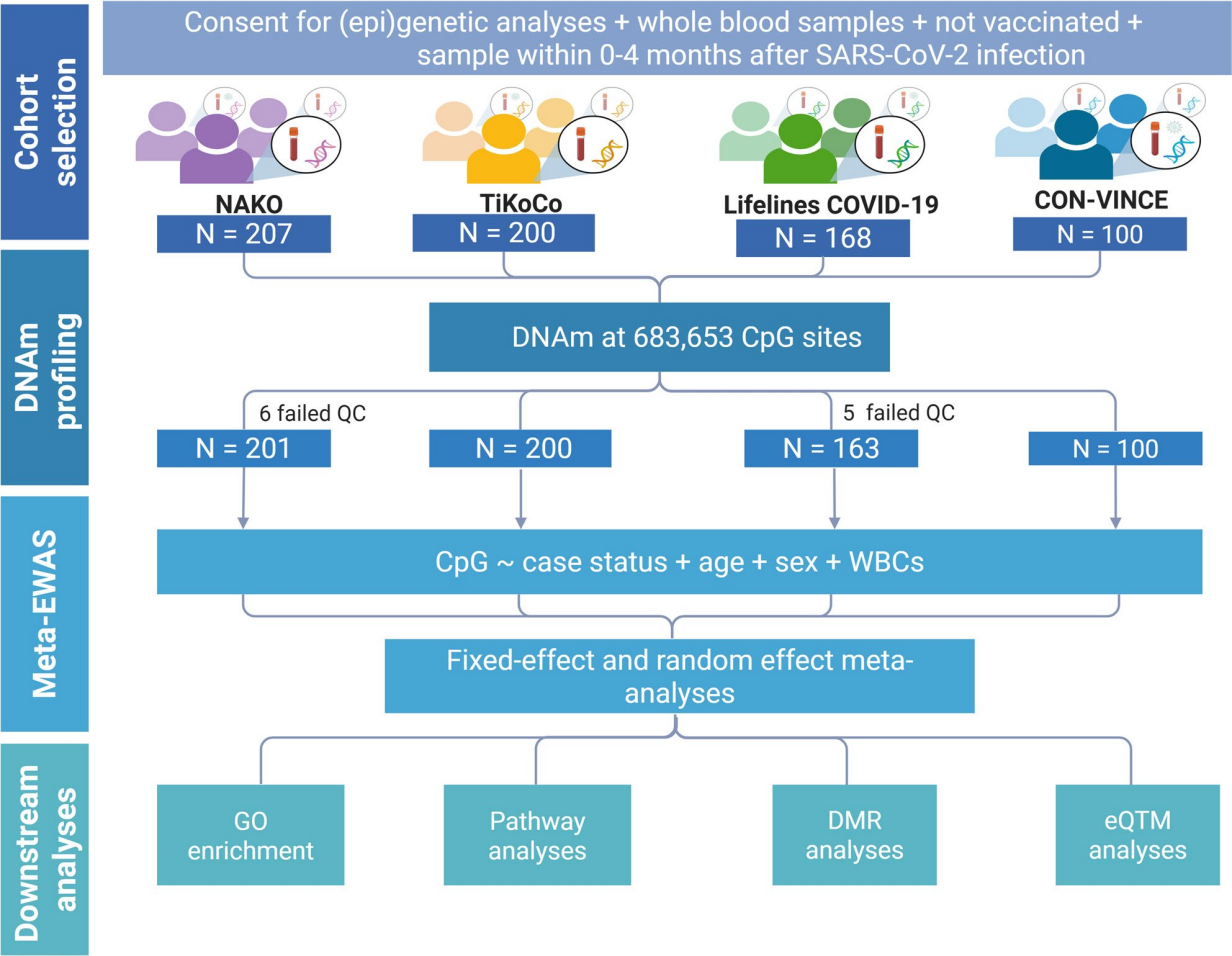
Overview of study design. Participants from four population-based cohorts were selected according to predefined criteria (light blue box). DNA was extracted from blood samples, and DNA methylation (DNAm) at ~ 850,000 CpG sites was measured using the Illumina EPIC array. DNAm data underwent preprocessing, quality control and harmonization across cohorts; 683,653 CpG sites were available for analysis after these steps. Epigenome-wide analyses were performed with DNAm as the outcome and case status as the exposure, adjusting for age, sex, and blood cell proportions. Study-level results were combined in fixed- and random-effect meta-analyses. Significant CpGs were carried forward to downstream analyses, including GO/pathway enrichment, differentially methylated region (DMR) analyses and expression quantitative trait methylation (eQTM) analyses. Created in BioRender. Waldenberger, M. (2026) https://BioRender.com/lzuyisr

Population-based cohorts with available blood and/or genomic DNA samples for DNAm profiling were identified within the framework of the European Orchestra consortium [14]: German National Cohort (NAKO) [15], COvid-19 National survey for assessing VIral spread by Non-affected CarriErs (CON-VINCE) [16, 17], The Lifelines Corona Research Initiative [18, 19], and Tirschenreuth Kohorte COVID-19 (TiKoCo) [20, 21] (Fig. 1). Participants from these cohorts were selected if they met the following inclusion criteria: (i) attended an examination where demographic/clinical data was collected and whole blood sampling was done, (ii) provided informed consent for (epi)genetic analyses, (iii) had (self-reported) data on COVID-19 testing within the last 4 months prior to the examination/blood draw, (iv) had not received a COVID-19 vaccine, and (v) were 18 years or older. Cases were identified as individuals with positive serology results and/or reporting positive COVID-19 testing (PCR, rapid test, self-test). Controls were selected from the pool of individuals with negative self-reported COVID-19 status or negative serology results; controls were matched to cases based on sex and age (see Supplemental Note 1 for further details).

### DNA methylation profiling and data processing

Whole blood or DNA samples from the selected cohorts were shipped to Helmholtz Munich for processing and DNA-methylation (DNAm) profiling. Samples meeting basic quality measures were used for DNAm analyses. For each sample from all included cohorts, 750 ng of genomic DNA was used for bisulfite conversion with the EZ-96 DNA Methylation Kit (Zymo Research, Orange, CA, USA). Methylation profiling was done following using the iScan platform (San Diego, CA, USA) and the Infinium MethylationEPIC BeadChip following standard protocols defined by Illumina. Initial quality control of the assay and generation of export data was done in GenomeStudio (v2011.1, RRID: SCR_010973) with Methylation Module version 1.9.0.

Quality control, preprocessing of the data and statistical analyses were performed in R v4.1.3 (R Core Team (2017). R: A language and environment for statistical computing. R Foundation for Statistical Computing, Vienna, Austria. URL https://www.R-project.org/).

Data was processed using the package minfi v1.40.0 [22] following the CPACOR pipeline [23], as described in [24]. In brief, raw intensities were read into R and background corrected. The cut-off for the detection rate in each study was determined individually, taking into account results from two quality control steps: (i) comparison of predicted sex, based on DNA methylation patterns, with reported sex to identify potential sample mislabelling, and (ii) assessment of median intensity values. The goal is to optimize the sample size while maintaining data quality, so that the cohorts’ range for this threshold was 80%−95% (see Suppl. Note 2 for additional details). Prior to normalization, samples failing sex prediction or falling below median intensity were removed. Likewise, probes with known issues were removed (cross-reactive, with minor allele frequency minor allele > 5% at the CG or the single base extension positions, and > 5% missing values); additionally, probes from EPICv1 not represented in the EPICv2 and probes in sex chromosomes were removed. Quantile normalization (QN, R package limma v3.50.3, RRID: SCR_010943 [25]) was performed as described in [24]; briefly, this was done separately on the signal intensities divided into the 6 probe types [23]. QN was performed for all samples together for the autosomes, and the transformed intensities were then used to generate methylation beta values: a measure from 0 to 1 indicating the percentage of cells methylated at a given locus. A total of 683,653 CpGs passed quality control measures across all profiled cohorts.

### Epigenome-wide association analysis (EWAS)

The association between DNA-methylation (DNAm) at 683,653 CpGs and SARS-CoV-2 infection (case/control) was examined in an epigenome-wide association study (EWAS) of differentially methylated positions (DMPs) using linear regression models in each cohort separately. As methylation levels in blood can be strongly influenced by leukocyte composition, we performed white blood cell deconvolution from bulk blood DNAm samples, using the R package FlowSorted.BloodExtended.EPIC and the function estimateCellCounts2() on the raw intensities and default parameters. This produced estimates for 12 cell types: neutrophils (Neu), eosinophils (Eos), basophils (Bas), monocytes (Mono), B naïve cells (Bnv), B memory cells (Bmem), T-helper CD4 + naïve cells (CD4nv), T-helper CD4 + memory cells (CD4mem), T regulatory cells (Treg), T-cytotoxic CD8 + naïve cells (CD8nv), T-cytotoxic memory CD8 + cells (CD8mem), and natural killer cells (NK) [26]. DNAm levels were defined as the dependent variable and case status as the predictor variable, where one model was run per CpG site. Variables included in the main model to address potential confounding were age, sex and 11 variables from the deconvoluted white blood cell proportions; analyses in NAKO additionally included a technical covariate (array version). A second model additionally adjusted for smoking (current smoker, former smoker, never smoker) and prevalent chronic disease (cardiovascular disease, lung disease, and metabolic disease; see Suppl. Note 1 for details on cohort-specific definitions of this variable). Analyses were done using the function lm() from the package stats v4.2.2.

Results were quality controlled using the R package QCEWAS v1.2–3; during this QC, results files are checked for data integrity and validity (e.g. no negative standard errors or p-values), cohort-specific outlier detection and removal of sex chromosomes [27]. Quantile–Quantile (Q–Q) plots and additional diagnostic plots (histogram for effect sizes and standard errors, volcano plot with distribution of effect sizes in relation to p-values and Manhattan plots) were generated to assess over/under-significance of the results and comparability of effect sizes. Conventional genomic inflation factors (λ) were calculated to evaluate inflation in each EWAS.

### Meta-analyses

Cohort-level results were meta-analyzed for 667,707 CpGs with available EWAS results across all cohorts using both fixed-effects (FE) and random-effects (RE) models in METAL (RRID: SCR_002013) [28]. The FE model combined study-level estimates under the assumption of a common underlying effect, weighting by the inverse of their variance. The RE model incorporated both within- and between-study variance (τ²), with the latter estimated using the DerSimonian–Laird method. For each CpG, measures of heterogeneity included the chi-squared statistic for heterogeneity (HetChiSq), its p-value (HetPVal), the I² statistic (0–100%), and τ², a measure of between-study variance.

Associations with consistent direction of effect across cohorts and meta-analysis p-values below the Bonferroni-corrected threshold (α = 0.05; *p* < 7.48 × 10⁻⁸) were considered statistically significant, while associations with *p* < 1 × 10⁻⁵ were considered suggestive. For CpG–case status associations showing substantial heterogeneity (I² > 70%), leave-one-out meta-analyses were conducted as sensitivity analyses to identify influential studies using the meta and dmetar R packages [29–31]. An additional sensitivity analysis was performed in a subset of participants with serology-confirmed case status (TiKoCo and CONVINCE; *N* = 292).

### Differentially methylated region (DMR) analyses

Differentially methylated regions (DMRs) represent genomic regions with consistently altered DNA methylation across multiple adjacent CpG sites. To complement the single-site differentially methylated position (DMP) analysis, DMR analyses were done with the meta-analyses results and at the cohort-level using the comb-p function (Enmix v1.38.01; dist.cutoff = 1000, seed = 1e-2 and bin.size = 310) [32]. Sensitivity analyses varying one parameter at a time using more stringent or lenient values were conducted (SA1 with seed = 1-e03, SA2 with d = 500, SA3 with b = 500, SA4 b = 500 and s = 1e-03). Coordinates from DMRs were converted to GRange objects and annotated using the R packages GenomicRanges (v1.54.01) [33] and the EPIC annotation dataset from chAMP (2.32.0) [34]. Meta-DMRs and cohort-level DMRs were compared by coordinate overlap and shared CpG probes to assess replication.

### Enrichment analyses and annotation

Gene ontology (GO) and KEGG pathway enrichment analyses (RRID: SCR_012773) were performed using the goMeth function from the missMethyl R package (v.1.32.1) [35]. Additional potentially relevant pathways were queried in WikiPathways (RRID: SCR_002134) [36]. To explore whether the identified genes showed cell type-specific expression, single-cell RNA-seq data from 18 sorted immune cell types was queried in the Human Protein Atlas [37, 38]. Annotation to regulatory regions and chromatin states was done using the PBMC Roadmap reference epigenome (E062) [39]. Association with other phenotypes was queried in the EWAS Catalog [40]. An overrepresentation analysis (ORA) was done using WebGestalt (RRID: SCR_006786) to explore enrichment of genes in Pathway Figure OCR (PFOCR) [41, 42]. The influence of genetic variants in the identified CpGs, namely the overlap with methylation quantitative trait loci (meQTL), was queried in the BIOS QTL database and GoDMC [43, 44].

### Expression quantitative trait methylation (eQTM) analyses

To investigate the functional relevance of these methylation changes, expression quantitative trait methylation (eQTM) analyses were done to identify associations between the complete set of identified CpG sites (DMP and DMR analyses) and gene expression using DNAm and RNA sequencing (RNA-seq) data from the KORA FF4 cohort (see Suppl. Note 3). After quality control, the data were available for 1,543 individuals. Gene expression probes within a 500 kb window surrounding significant CpGs were analyzed using the MatrixEQTL package (version 2.3) [45]. Linear models were adjusted for age, sex, measured white blood cell proportions (neutrophils, monocytes, basophils, and eosinophils), and technical variation, with multiple testing controlled by Bonferroni correction.

## Results

### Cohort description

Table 1 shows population characteristics for the 675 participants from 4 cohorts included in this study. The cohorts largely consisted of similar age distributions and sex ratios. The average age ranged from 41 to 57 years, with a balanced representation of males and females across cases and controls. Smoking prevalence between cases and controls was similar. No significant differences were found in BMI, smoking groups or prevalence of chronic disease (cardiovascular, metabolic and lung disease) between both groups across all cohorts. Twelve blood cell types were deconvoluted from DNAm data and estimated for all participants from the studied cohorts; the only statistically significant difference observed was higher levels of Basophils in cases compared to controls in NAKO (*p* = 0.009).

**Table 1:** Population descriptives from included cohorts

Cohort	NAKO			TiKoCo			Lifelines COVID-19			CONVINCE		
	Case	Control	p	Case	Control	p	Case	Control	p	Case	Control	p
*Demographic variables*												
n	102	105		100	100		81	80		50	50	
age (mean (SD))	51.14 (10.73)	51.98 (11.99)	0.595	56.10 (14.35)	56.44 (14.61)	0.868	52.95 (9.23)	52.88 (8.61)	0.957	41.74 (13.94)	40.90 (13.90)	0.763
*sex (%)*												
male	52 (51.0)	60 (57.1)	0.453	50 (50.0)	50 (50.0)	1	31 (38.3)	31 (38.8)	1	23 (46.0)	23 (46.0)	1
female	50 (49.0)	45 (42.9)		50 (50.0)	50 (50.0)		50 (61.7)	49 (61.3)		27 (54.0)	27 (54.0)	
BMI (mean (SD))	25.96 (4.77)	25.71 (4.04)	0.683	27.56 (4.59)	28.22 (5.50)	0.36	26.98 (4.16)	27.36 (4.52)	0.580	26.92 (5.36)	26.68 (4.81)	0.816
*smoking (%)*												
never	56 (54.9)	42 (40.0)	0.1	56 (56.0)	54 (54.0)	0.163	28 (34.6)	37 (46.2)	0.185	36 (72.0)	28 (56.0)	0.201
former	29 (28.4)	40 (38.1)		34 (34.0)	27 (27.0)		29 (35.8)	19 (23.8)		8 (16.0)	15 (30.0)	
current	17 (16.7)	23 (21.9)		10 (10.0)	19 (19.0)		< 10 (< 8)	< 10 (< 8)		6 (12.0)	7 (14.0)	
Chronic disease (%)												
no	45 (44.1)	44 (41.9)	0.856	67 (67.0)	67 (67.0)	0.84	60 (74.1)	54 (67.5)	0.653	43 (86.0)	44 (88.0)	1
yes	57 (55.9)	61 (58.1)		32 (32.0)	31 (31.0)		18 (22.2)	22 (27.5)		7 (14.0)	6 (12.0)	
NA	0	0		1 (1.0)	2 (2.0)		3 (3.7)	4 (5.0)				
*Estimated blood cell proportions*												
Bas (mean (SD))	0.01 (0.01)	0.00 (0.01)	0.009	0.01 (0.01)	0.01 (0.01)	0.682	0.01 (0.01)	0.01 (0.01)	0.812	0.01 (0.01)	0.00 (0.01)	0.12
Bmem (mean (SD))	0.02 (0.01)	0.02 (0.01)	0.681	0.02 (0.02)	0.02 (0.01)	0.964	0.02 (0.01)	0.02 (0.01)	0.434	0.03 (0.04)	0.02 (0.02)	0.681
Bnv (mean (SD))	0.03 (0.02)	0.02 (0.02)	0.098	0.02 (0.02)	0.02 (0.01)	0.086	0.02 (0.02)	0.02 (0.01)	0.545	0.02 (0.02)	0.02 (0.02)	0.827
CD4mem (mean (SD))	0.11 (0.04)	0.11 (0.05)	0.424	0.10 (0.04)	0.10 (0.04)	0.244	0.09 (0.04)	0.08 (0.04)	0.19	0.11 (0.04)	0.10 (0.04)	0.489
CD4nv (mean (SD))	0.07 (0.04)	0.06 (0.04)	0.025	0.05 (0.04)	0.05 (0.04)	0.373	0.07 (0.04)	0.06 (0.04)	0.209	0.07 (0.04)	0.07 (0.04)	0.821
CD8mem (mean (SD))	0.06 (0.05)	0.06 (0.06)	0.819	0.06 (0.05)	0.06 (0.05)	0.863	0.06 (0.05)	0.06 (0.05)	0.91	0.07 (0.05)	0.06 (0.05)	0.427
CD8nv (mean (SD))	0.01 (0.02)	0.01 (0.02)	0.401	0.01 (0.01)	0.01 (0.01)	0.479	0.01 (0.02)	0.01 (0.02)	0.623	0.02 (0.02)	0.02 (0.02)	0.227
Eos (mean (SD))	0.01 (0.01)	0.01 (0.01)	0.556	0.01 (0.02)	0.01 (0.02)	0.698	0.01 (0.02)	0.01 (0.02)	0.572	0.01 (0.02)	0.01 (0.02)	0.999
Mono (mean (SD))	0.08 (0.02)	0.08 (0.03)	0.915	0.08 (0.02)	0.08 (0.02)	0.373	0.08 (0.02)	0.08 (0.02)	0.157	0.07 (0.02)	0.07 (0.03)	0.66
Neu (mean (SD))	0.51 (0.11)	0.54 (0.12)	0.066	0.53 (0.09)	0.54 (0.09)	0.284	0.49 (0.09)	0.52 (0.10)	0.162	0.50 (0.11)	0.50 (0.10)	0.832
NK (mean (SD))	0.06 (0.02)	0.06 (0.03)	0.88	0.05 (0.02)	0.06 (0.02)	0.236	0.06 (0.03)	0.07 (0.03)	0.701	0.05 (0.02)	0.06 (0.03)	0.171
*Assessment of SARS-CoV-2 infection*												
Methodology	Self-reported positive test			Self-reported positive test and serology			Self-reported (physician-diagnosed) infection (until 12–2020) and positive test (from 12–2020 onwards)			Self-reported positive test and serology		
Period of data collection	01/05/2020 to 01/10/2020 and 01/09/2022 to 01/01/2023			06/29/2020 to 07/17/2020			30/03–2020 to 12/01/2022			05/2020 to 06/2021		
Period of blood sample collection for DNAm	01/09/2020 to 01/04/2023			06/29/2020 to 07/17/2020			04/2020 to 03/2022			05/2020 to 06/2021		
*Serology*												
*IgG_status (%)*												
Negative	NA	NA		100 (100.0)	0 (0.0)		NA	NA		8 (16.0)	50 (100.0)	< 0.001
Positive	NA	NA		0 (0.0)	100 (100.0)		NA	NA		40 (80.0)	0 (0.0)	
Borderline	NA	NA		0 (0.0)	0 (0.0)	< 0.001	NA	NA		2 (4.0)	0 (0.0)	
IgA_status (%)												
Negative	NA	NA		NA	NA		NA	NA		18 (36.0)	47 (94.0)	< 0.001
Positive	NA	NA		NA	NA		NA	NA		29 (58.0)	3 (6.0)	
Borderline	NA	NA		NA	NA		NA	NA		3 (6.0)	0 (0.0)	

Summary of demographics, blood cell proportions, and serology-based status of participants from the four cohorts. Continuous variables are presented as mean (SD) with p-values from t-tests; categorical variables are shown as counts (%) with p-values from chi-squared tests

### Epigenome-wide association analysis (EWAS)

Epigenome-wide association analyses were conducted in all studies individually (Supplemental Tables 1–4, Supplemental Figs. 1–4). EWAS results from all population-based cohorts showed comparable effect sizes and reasonable precision-sample size ratios (Suppl. Figure 5); genomic inflation values ranged from λ = 0.931 to λ = 1.007, so no genomic control correction was done. No deviations from expected precision in the obtained estimates was observed (Suppl. Figures 1–5). Two CpG sites, cg13452062 and cg03607951, both annotated to *IFI44L*, reached Bonferroni-corrected significance in the Lifelines study. No significant signals were observed in the CONVINCE, NAKO, or TiKoCo studies.

### Meta-EWAS results

**Fig. 2:**
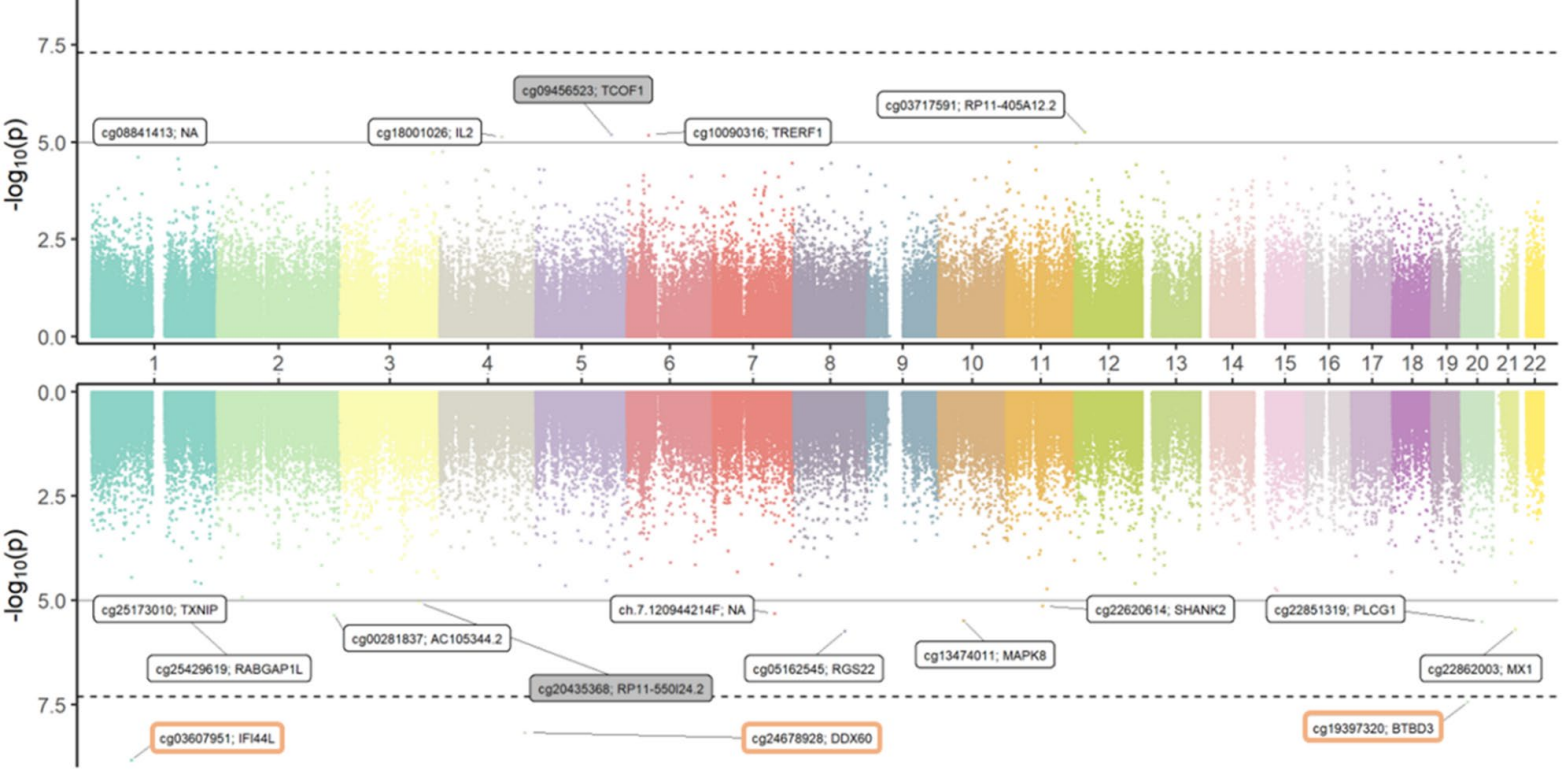
Associations between genome-wide CpG methylation levels and case status from meta-EWAS Manhattan plot of meta-EWAS results for CpG methylation and case status. The X-axis shows genomic position by chromosome; the Y-axis shows –log₁₀ p-values from the IVW FE meta-analysis. The upper half of the plot represents CpG sites with positive effect estimates, while the lower half shows CpG sites with negative effect estimates. Each point represents a CpG, colored by chromosome. 16 probes significant at the suggestive threshold (*p* < 1 × 10⁻⁵) are labeled, with Bonferroni-significant CpGs highlighted in orange. Grey-labeled CpGs (cg09456523, cg20435368) showed inconsistent effect directions across cohorts and were not considered robust

**Fig. 3:**
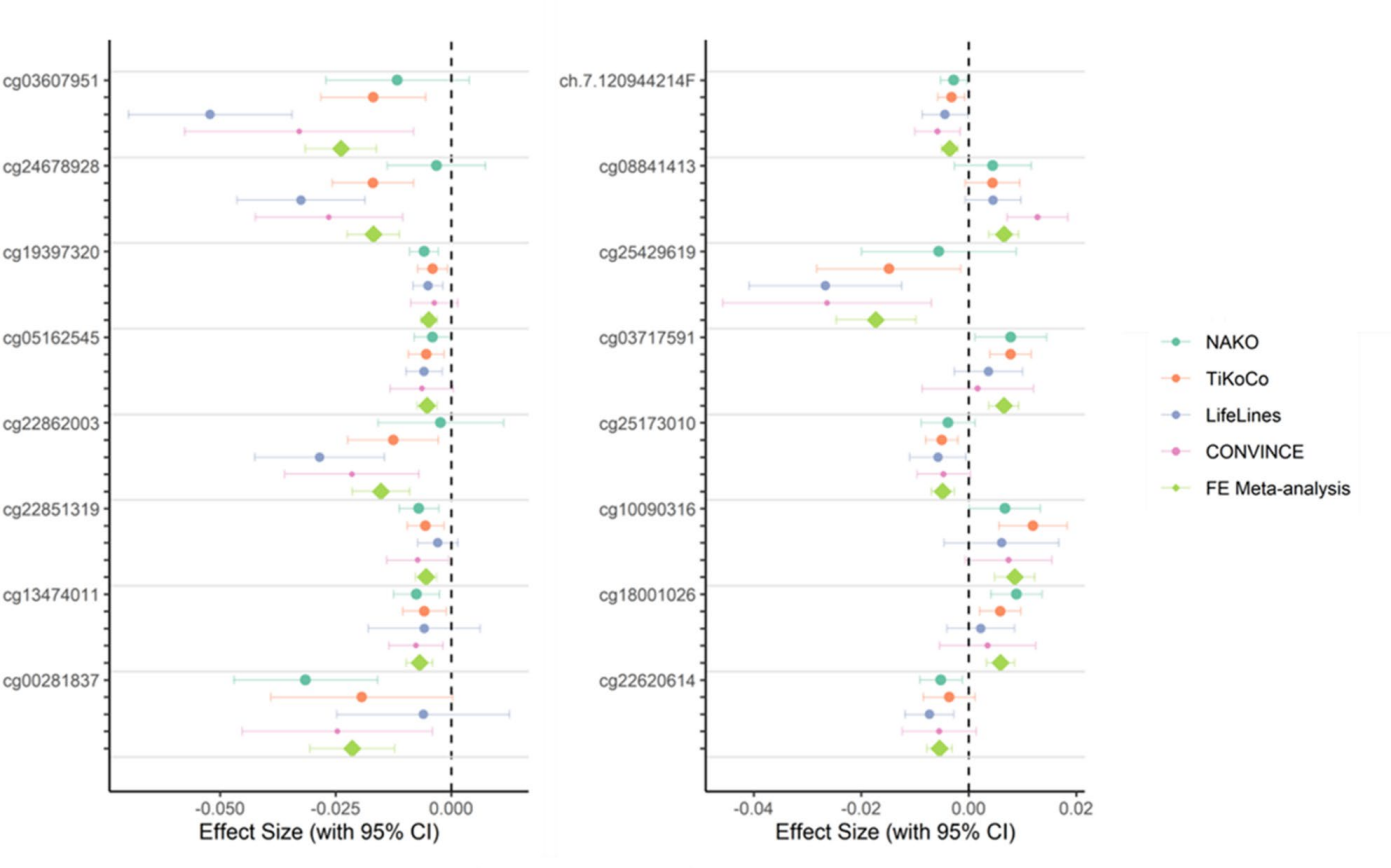
Forest plots of CpG~case status associations. Circles (cohorts) show effect sizes with 95% CI, scaled by sample size; diamonds show pooled FE meta-analysis estimates. Probes are ordered by significance

Differences in DNAm levels between individuals who had a SARS-CoV-2 infection in the prior 4 months and controls were identified in 16 CpGs annotated to 12 different genes (10 protein-coding and 2 encoding for long non-coding RNA transcripts) at suggestive threshold (p < 1 × 10⁻⁵); 3 of these CpGs were significant after Bonferroni correction: cg03607951, cg24678928 and cg19397320 (Fig. 2). Notably, 75% of these identified CpGs were hypomethylated in individuals who had a SARS-CoV-2 infection (Table 2), and cohort-specific estimates were consistent in direction and comparable in magnitude (Fig. 3). Most of these CpGs are located in promoter regions (7 CpGs [43.7%] in TSS1500, 1 [6%] in 1 st exon, and 2 [12.5%] in 5’ UTR), followed by 6 in non-promoter regions (5 CpGs [31.2%] in IGR) and 1 in the gene body (1 CpG [6.2%]) (Table 2). EWAS summary statistics are provided in Suppl. Table 5. DNAm at cg03607951 (annotated to *IFI44L*) shows the strongest association with case status (effect = −0.024 [−0.032, −0.016], *p* = 1.50E-09); the direction of this effect was consistent across studies (Fig. 3). Although heterogeneity was high given the variability in the effect sizes observed across cohorts (I^2^ = 78.9%), low variability between studies was observed (Suppl. Table 6). Similar results were observed for cg24678928 (*DDX60*; effect = −0.017 [−0.023, −0.011], *p* = 6.37E-09, I^2^ = 76.3%). In the case of cg03607951, lowest heterogeneity and smallest pooled effect sizes were observed when Lifelines was omitted from analysis (I^2^ = 2% and effect = −0.02 [−0.03,−0.01]; Suppl. Figure 6). The omission of NAKO had a similar effect in reducing heterogeneity in the association for cg24678928 (I^2^ = 47%; Suppl. Figure 7). In the case of cg19397320 (*BTBD3*; effect = −0.005 [−0.006, −0.003], *p* = 6.21E-08), no heterogeneity was observed and thus estimates from the random-effects model were the same. All the identified associations were slightly attenuated by adjusting for prevalent chronic disease and smoking, but the estimated effect sizes in both models were highly correlated (Spearman’s rank corr = 0.99, *p* = 1.037E-05) and remained significant at *p* < 1e-03 (Suppl. Table 6). Effect sizes estimated at the epigenome-wide level in both models also showed a high correlation (Pearson’s corr = 0.948, *p* < 2.2E-16; full summary statistics from model further adjusting for smoking and prevalent chronic disease in Suppl. Table 7). Similarly, the effects estimated in the sensitivity analysis including only cohorts with a serology-based case definition (TiKoCo and CONVINCE, *N* = 292) robustly replicated those of the main analyses with lower heterogeneity (Suppl. Table 8).

**Table 2:** Differentially methylated positions (DMPs) associated with case status in the meta-EWAS

CpG	Genomic location	Gene	Chr	Dir	FE Meta-analysis					RE Meta-analysis		
					Effect	SE	pval	I^2^	HetPVal	Effect	SE	Pvalue
										ARE	ARE	ARE
cg03607951^#^	TSS1500	*IFI44L*	1	----	−0.024	0.004	1.50E-09	78.9	0.003	−0.027	0.009	0.0027
cg24678928^#^	TSS1500	*DDX60*	4	----	−0.017	0.003	6.73E-09	76.3	0.005	−0.019	0.006	0.0024
cg19397320*	1stExon	*BTBD3*	20	----	−0.005	0.001	3.71E-08	0	0.83	−0.005	0.001	3.71E-08
cg05162545*	TSS1500	*RGS22*	8	----	−0.005	0.001	1.84E-06	0	0.907	−0.005	0.001	1.84E-06
cg22862003^#^	TSS1500	*MX1*	21	----	−0.015	0.003	2.00E-06	62.3	0.047	−0.016	0.005	0.003
cg22851319*	TSS1500	*PLCG1*	20	----	−0.005	0.001	3.12E-06	0	0.557	−0.005	0.001	3.12E-06
cg13474011*	IGR	*MAPK8*	10	----	−0.007	0.001	3.24E-06	0	0.951	−0.007	0.001	3.24E-06
cg00281837	IGR	*AC105344.2*	2	----	−0.021	0.005	4.44E-06	31.1	0.225	−0.021	0.006	0.0002
ch.7.120944214 F*	IGR		7	----	−0.004	0.001	4.81E-06	0	0.635	−0.004	0.001	4.81E-06
cg08841413	IGR		1	++++	0.007	0.001	5.12E-06	52	0.1	0.007	0.002	0.0017
cg25429619^#^	5’UTR	*RABGAP1L*	1	----	−0.017	0.004	5.32E-06	42.2	0.158	−0.018	0.005	0.0005
cg03717591*	IGR	*RP11-405A12.2*	12	++++	0.007	0.001	5.52E-06	0	0.529	0.007	0.001	5.52E-06
cg25173010*	TSS1500	*TXNIP*	1	----	−0.005	0.001	5.91E-06	0	0.964	−0.005	0.001	5.91E-06
cg10090316*	5’UTR	*TRERF1*	6	++++	0.009	0.002	6.57E-06	0	0.646	0.009	0.002	6.57E-06
cg18001026	TSS1500	*IL2*	4	++++	0.006	0.001	7.45E-06	2.5	0.38	0.006	0.001	1.14E-05
cg22620614*	Body	*SHANK2*	11	----	−0.005	0.001	7.52E-06	0	0.752	−0.005	0.001	7.52E-06

Sixteen CpG probes associated with prior SARS-CoV-2 infection (p < 1 × 10⁻⁵) in a meta-analysis of 675 samples from four cohorts. Bonferroni-significant CpGs (p < 7.48 × 10⁻⁸, FE) are in bold; those also significant in RE are marked with *. CpG: probe; Genomic location: Genomic annotation categories in relation to regulatory features (TSS200: CpG located within 200 bp upstream of transcription start site (TSS), TSS1500: CpG 200–1500 bp upstream of TSS, 5’UTR: located in 5’ untranslated region, 1 st exon: CpG located in first coding exon, Body: within gene body, IGR: intergenic region); I^2^: statistic measuring heterogeneity on scale of 0–100%; HetPVal: P-value for heterogeneity statistic. Previously reported CpG sites associated with COVID-19 were labeled as “#”.

### DMR results

Table 3 shows 21 DMRs from the meta-EWAS (Sidak *p* < 0.05), composed by 101 CpG sites and overlapping 10 genes and 2 pseudogenes; eight are in proximity to promoter regions, followed by nine in non-promoter regions. All DMRs were hypomethylated, in full concordance with the DMP effects observed across cohorts (Suppl. Table 9). The top two CpGs in the DMP analyses were also identified in the DMR analyses but lacked enough adjacent CpGs to meet DMR criteria. Sensitivity analyses using different parameters to call DMRs produced 8 to 30 DMRs (Suppl. Table 10); eight DMRs were consistently identified across all analyses (Suppl. Table 11). Of note, two of the sensitivity analyses allowing for larger DMRs identified an additional *IFI44L*-annotated CpG (Suppl. Table 12), thus producing a 3-CpG-DMR overlapping DMP results.

In the DMR analyses conducted across four cohorts, a total of 51 distinct DMRs were identified (20 in NAKO, 13 in TiKoCO and Lifelines each, 5 in CON-VINCE; Suppl. Table 13). Five of the meta-DMRs were replicated in one cohort each: DMR3 and DMR21 in TiKoCo, DMR5 and DMR18-*THRB* in NAKO, and DMR10-*PARP9* in Lifelines (Suppl. Table 14). There was no overlap of Sidak significant DMRs across the four cohorts (Suppl. Table 15).

**Table 3:** Differentially methylated regions (DMRs) associated with case status in the meta-EWAS

DMR ID	DMR description	*N* probes	Sidak pval	Overlapping CpGs	Genomic annotation
1*	chr13:24519920–24,520,508	5	3.05E-10	cg02002217;cg08405447;cg11450541;cg16014862; cg18976044	[IGR]; *ANKRD20A19P* [body]
2*	chr17:79495006–79,495,468	7	1.69E-08	cg09084279;cg13468858;cg13700073;cg15774065; cg16507479;cg18919720;cg20932150	*FSCN2* [TSS200, TSS1500, 5’UTR]
3*	chr13:23309689–23,310,226	7	2.35E-08	cg01863042;cg03042692;cg05215994;cg08083251; cg15973954;cg20395040;cg26361286	[IGR]
4*	chr7:2802554–2,803,066	8	7.66E-07	cg00947599;cg03459839;cg05793240; cg12102607;cg12444411;cg20884522;cg23285459; cg23333125	*GNA12* [TSS200, TSS1500, body]
5*	chr3:127634188–127,634,460	6	6.32E-06	cg05768427;cg08488841;cg10961186;cg12311882; cg13859958;cg14701491	[IGR]
6	chr14:70690287–70,690,705	6	4.94E-05	cg04204356;cg08322580;cg21484985;cg23442650; cg23618713;cg26146732	[IGR]
7*	chr1:234367443–234,367,587	3	8.10E-05	cg09119043;cg23445321;cg24723561	*SLC35F3* [body]
8*	chr19:45885800–45,886,079	4	0.000153258	cg04683509;cg16655626;cg21219373;cg23397216	*PPP1R13L* [body]
9	chr8:102236522–102,236,832	5	0.000166719	cg02168442;cg03250224;cg13211302;cg16058797; cg23042086	[IGR]
10*	chr3:122281939–122,282,158	3	0.000198913	cg00959259;cg07815522;cg08122652	*PARP9* [5’ UTR]
11	chr6:2876762–2,877,000	4	0.000279189	cg03500459;cg07704716;cg11678426;cg20958467	*SERPINB9P1* [TSS200, TSS1500]
12	chr2:121338498–121,338,608	3	0.000524689	cg03792788;cg07218647;cg09408902	
13	chr12:131303016–131,303,248	4	0.000653375	cg05407909;cg10832093;cg11011512;cg20050828	*STX2* [body]
14	chr6:134436460–134,436,741	3	0.001424293	cg07710266;cg12383699;cg14997321	[IGR]
15	chr4:74847710–74,848,017	7	0.001831057	cg02530824;cg05509609;cg06834998;cg13126871; cg15398841;cg16072462;cg21043213	*PF4* [TSS200, TSS1500, 1 st exon]
16	chr10:5406890–5,407,120	7	0.003325681	cg01134643;cg11218842;cg12428514;cg15671083; cg17158564;cg26644395;cg27349333	*UCN3* [TSS200, 1 st exon]
17	chr7:156735383–156,735,657	4	0.004911592	cg03930209;cg17604655;cg22871949;cg27539527	
18	chr3:24536765–24,536,890	4	0.005301857	cg04928005;cg25829666;cg26139133;cg26146027	*THRB* [TSS1500]
19	chr11:69286231–69,286,353	5	0.009814467	cg00695387;cg13293618;cg15442037;cg18498241; cg21408915	[IGR]
20	chr1:95008041–95,008,118	3	0.013555894	cg03460527;cg09549015;cg25893275	*F3* [TSS1500]
21	chr12:7781093–7,781,289	3	0.020072455	cg10578777;cg14906510;cg25828445	[IGR]

DMR ID: Identifier assigned to each differentially methylated region detected in the meta‑EWAS; DMR description — Genomic coordinates of the region (chromosome, start, end) summarizing the span of the DMR; N probes — Number of Illumina EPIC CpG probes constituting the region; Sidak pval: Sidak‑adjusted region‑level p‑value; Overlapping CpGs: individual CpG probe IDs located within the DMR boundaries; genomic annotation: annotation of the DMR based on Illumina EPIC definitions, including annotated gene and gene context (TSS200, TSS1500, 5′UTR, 1 st Exon, Body, 3′UTR, IGR). DMRs replicated across sensitivity analyses are marked with a star (*) next to their ID

### Enrichment analyses and annotation

The set of 117 CpG sites identified in either DMP- or DMR-analyses were annotated to 22 protein-coding genes (Suppl. Table 13). Even though no statistically significant enrichment of gene-sets in the GO or KEGG pathway databases were found, these analyses are helpful in contextualizing our findings in relation to biological functions. Top GO terms are related to response to viral infections (Suppl. Figure 8 and Suppl Table 16). Forty-seven KEGG pathways were represented in this list of CpGs (Suppl. Table 17), which included pathways related to immune and inflammatory diseases. Likewise, *IFI44L*, *MX1* and *MAPK8* were assigned to pathways involved in downstream molecular events triggered by SARS-CoV-2 infection (WikiPathways WP5115). Five genes showed immune-cell specific expression (Suppl. Table 18); for example, *THRB* had higher expression in basophils and naïve B-cells, *SLC35F3* expression was specific to dendritic cells and *PF4* specific to neutrophils. Additional analyses using WebGestalt showed a high overlap between the gene set identified in these analyses and those identified in an epigenetic signature of Systemic Lupus Erythematosus and HIV immune control (Suppl. Figure 9; Suppl. Table 19). meQTL were found n GoDMC for a few of the identified CpGs (e.g. cg25173010 in *TXNIP* and cg22862003 in *MX1*). The set of 16 identified CpGs were annotated to six chromatin states (Suppl. Table 20), of which TssBiv was suggestive of enrichment (OR = 7.76, *p* = 0.04; Suppl. Figure 10).

### Association between DNA methylation and gene expression

Several of the CpGs hypomethylated in cases were associated with expression of nearby genes. We identified 49 CpG–transcript pairs in whole blood in an eQTM analysis using RNA-seq and methylation data from KORA FF4 (Suppl. Table 21). Most CpG-gene transcript pairs had a negative association (69.39%, average effect size of − 1.92), where hypomethylation was associated with increased gene expression; the remaining pairs showed increasing methylation levels with higher gene expression (average effect size of 0.47). Cg03607951 **(***IFI44L***)**, the top CpG from the DMP analysis, showed strong negative associations with its gene transcript (Suppl. Figure 11 A). Notably, all seven CpGs in DMR4 (*GNA12*) were negatively associated with its gene (Suppl. Figure 11B); likewise, all 3 CpGs in DMR10 (*PARP9*) were associated with multiple transcripts (*PARP9*, *PARP14* and *DXTL30*) and positively associated with *KPNA1* (Suppl. Table 21; Suppl. Figure 11 C).

## Discussion

We conducted the largest EWAS to date assessing DNAm changes up to four months after SARS-CoV-2 infection in 675 individuals from four population-based European cohorts within the ORCHESTRA Consortium [14]. DNAm changes were identified in 16 DMP and 21 DMRs, most of which were located in promoter regions or body of protein-coding genes; most DMPs and all DMRs were hypomethylated in cases vs. controls. Three DMPs reached genome-wide significance after Bonferroni-correction (*p* < 7.48E-08): the strongest association was observed for cg03607951 (*IFI44L*), followed by cg24678928 (*DDX60*) and cg19397320 (*BTBD3*). The top two DMPs were also identified in DMR sensitivity analyses, supporting the robustness and complementarity of the DMR analyses. We also demonstrate that SARS-CoV-2–associated DNAm alterations at both the DMP and DMR levels are linked to transcriptional differences, with downstream analyses indicating these regions are involved in immune response pathways and pro-inflammatory processes.

Cell deconvolution methods were applied to estimate WBC proportions across cohorts; no significant differences in their distribution were observed between cases and controls, except for basophil levels in NAKO. Two prior studies on DNAm changes 3–6 months post-infection also found no evidence of altered immune system composition in individuals with mild infection [11, 13], in contrast to significant changes correlated with COVID-19 severity observed during the acute phase of infection [1, 2]. In line with this and our WBC-adjusted analyses, most genes mapped to the top DMP/DMR associations did not show cell-type-specific expression in the HPA datasets. Adjusting for smoking and prevalent chronic disease attenuated the estimated effects, but the associations remained robust. This was expected, as both smoking and chronic disease are known risk factors for severe COVID-19 [46]. Furthermore, recent evidence suggests that smoking has short- and long-term effects mediated by DNAm on immune regulation that persist after smoking cessation [47]. Despite the high heterogeneity observed in two of the three strongest CpG-case status associations, the direction of effect was consistent across studies, and the associations remained robust in the leave-one-out and serology-based sensitivity analyses.

Our DMP and DMR results are in line with epigenetic changes seen in severe COVID-19 during the acute phase of infection [1–7]. The majority of probes associated with case status in this study exhibited lower DNA methylation levels, consistent with hypomethylation associated with severe COVID-19 during early disease [1, 4, 5] and three to twelve months post-infection [9, 11, 48]. Several interferon-stimulated genes (ISG) identified in the DMP analyses –namely *IFI44L*, *MX1* and *DDX60 –* have been consistently identified in severe [1–3, 5] and early mild COVID-19 [6]. *IFI44L* is involved in response to type I interferon (IFN), a key pathway in COVID-19 disease development and severity [49–51]. IFN are signalling cytokines involved in the regulation of the immune response to both viral and bacterial infections [52] and autoimmune disease [53]. *MX1* (MX Dynamin Like GTPase 1) is an interferon-induced gene (ISG) encoding for a GTPase that inhibits viral replication; genetic variation in *MX1* is associated with differential risk to developing severe COVID-19 [54] and its differential methylation is linked to disease progression [55]. Our DMP and eQTM results are in line with previous reports of hypomethylation in *IFI44L* both during acute infection and persisting for months thereafter [3, 8, 11]. Hypomethylation of *MX1* was identified across COVID-19 severity groups [2, 6], was specific to COVID-19 patients in comparison to patients with other respiratory diseases [4, 5]) and was still present at 12 months after infection [8, 10]. In addition to cg03607951-*IFI44L* and cg22862003-*MX1*, cg24678928 (*DDX60*) and cg25429619 (*RABGAP1L*) were hypomethylated in COVID-19 patients during the acute-phase of disease [5]; likewise, altered DNAm in cg24678928-*DDX60* and cg22862003-*MX1* was observed up to 3 months post-infection [11]. *DDX60* (DExD/H-Box Helicase 60), encodes for an RNA helicase acting as an antiviral factor involved in the interferon inducible response to viral infection, and it was included in DMP signatures in COVID-19 cases [5, 56]. Interestingly, hypomethylation at cg24678928-*DDX60* was correlated with worse quality of life in convalescent COVID-19 patients with Post-Acute COVID-19 Syndrome (PASC) [57].

These DMPs partially overlap DNAm changes in autoimmune and inflammatory disease [8, 11] – e.g. systemic lupus erythematosus [58], primary Sjögren’s Syndrome [59] and rheumatoid arthritis [60] – but also feature in interferon response to COVID-19 [61]. Importantly, individuals with autoimmune disease were excluded from analyses in cohorts where this information was available (see Supplemental Note 1). This points to shared dysregulation of the innate immune system [5, 6], a cross-talk between acute COVID-19 pathogenesis and autoimmune disease [62, 63], and further supports DNAm of ISGs to be involved in long-term dysregulation [8].

DMR analyses identified ten additional protein-coding genes associated with case status. Of these, DMRs in *PARP9* and *GNA12* show the strongest and most robust association with case status, as seen across sensitivity analyses, replication in at least one cohort, and supportive eQTM data. Although expressed in immune cells, their expression is not cell-type-specific. The location of DMR4-*GNA12* (in proximity to TSS) and DMR10-*PARP9* (5’ UTR) suggests these changes might regulate gene expression. In line with this, our eQTM results show hypomethylation across CpGs within DMR10 correlates with higher expression of several genes (*PARP9*, *PARP14*, *DTX3L*), and hypomethylation at DMR4 with expression of *GNA12*. PARP9-DT3XL is an interferon-induced module crucial to regulating PARP14 activity in a cascade of antiviral signaling [64], and *GNA12* encodes a G-protein subunit in signaling hub involved in cancer progression, vascular dysfunction and inflammation [65]. Our *PARP9* DMR and eQTM results replicate observations in COVID patients versus non-COVID respiratory controls [4]; likewise, DMR4 is in proximity to a DMP identified across COVID severity groups [6] and to two DMRs persistently hypomethylated one year post-infection [48]. Interestingly, this DMR almost perfectly replicates the COVID-19-specific DMR reported in [56]. Hypomethylation of *GNA12* and its corresponding increased gene expression have been reported one year post-infection in hospitalized patients [48]. DMR18 (*THRB*) was also replicated in at least one cohort and across all sensitivity analyses but SA1 (most strict seed value). *THRB* encodes for a receptor involved in thyroid-hormone signaling in metabolism and endocrine homeostasis, while its expression in immune cells points to endocrine-immune cross-talk [66]. Additional DMRs mapped to genes involved in endocrine-metabolic dysregulation and stress (*UCN3*, *SLC35F3*), as well as in inflammatory signaling (*FSCN12*, *GNA12*, *PPP1R13L*) and platelet activation and coagulation (*PF4*, *F3*, *STX2*) (Suppl. Table 22). Similar pathways were identified in a study on differential DNAm in mucosal nasal tissue of COVID-19 patients during the acute infection phase [67]. This study is the first to report persistent differential DNAm in these genes months after a SARS-CoV-2 infection.

We identified biologically plausible changes at the DMP and DMR level in whole blood of mild cases up to four months post-infection, with independent eQTM data supporting the potential functional impact in gene regulation of these subtle DNAm changes. Our findings suggest that subtle but persistent DNAm changes contribute to long-term epigenetic remodeling following a SARS-CoV-2 infection that overlaps pathways involved in metabolic, inflammatory and autoimmune disease. Other changes we identified in intergenic regions and pseudogenes are less directly related to specific molecular pathways, but potentially reflect widespread changes in the epigenome [68]. Despite multiple levels of heterogeneity in studies assessing long-term epigenetic changes (populations assessed, follow-up time and disease severity groups), DNAm changes at the DMP- and DMR-level partially replicate those observed in multiple independent studies. Our findings suggest some epigenetic injuries acquired during infection are persistent months after infection, although the epigenetic signature reported here is associated with mild infection in the general population and is therefore more subtle than the large effects observed during severe infections in hospitalized patients. A recent review on DNAm in persistent COVID-19 suggests infection alters the epigenetic landscape [69], with these changes potentially contributing to the molecular signature of long COVID [70].

This study is the largest multi-cohort cross-sectional EWAS in the months following a SARS-CoV-2 infection in population-based cohorts. Associations were consistent across multiple European cohorts, and the inclusion of non-vaccinated individuals allowed to control for confounding by vaccination-induced changes. Unlike earlier studies detecting large effects in small sample sizes, this study was designed to detect small sized effects (Cohen’s d = 0.2), which enabled the identification of less pronounced DNAm changes months after infection. These findings suggest that subtle but persistent epigenetic changes in blood mirror alterations in well-described immune and inflammatory pathways, help further understand the multi-systemic nature of long-term biological dysregulation following SARS-CoV-2 infection and offer initial insights into potential biomarkers for risk stratification or therapeutic intervention.

This study has several limitations. Time between infection and blood sampling (DNAm) was variable within and across cohorts, which contributed to case heterogeneity and is an important limitation inherent to cohorts’ study designs. Case definition in each cohort relied on differing SARS-CoV-2 detection methods (test-based or self-reported), so it may have been affected by recall and misclassification bias; however, sensitivity analyses using a serology-based case definition in a subset of the participants produced same conclusions. NAKO and Lifelines were identified as likely influential studies, potentially due to larger sample sizes and varying case definitions, yet leave-one-out sensitivity analyses produced similar results. Although inflation at the study-level was not significant, residual confounding is inherent to observational studies like this one. Beyond cohort heterogeneity, underlying inter-individual variation in disease severity/symptomatology and recovery cloud the interpretation of the results in terms of molecular mechanisms and pathophysiology. Additional limitations include limited coverage of the epigenome by using array-based methods, assessing autosomes only and profiling mixed white blood cell populations instead of other potentially more informative tissues [67, 71]. Study designs across cohorts did not allow for longitudinal analyses, assessment of concomitant gene expression or genetic variation, or the examination of epigenetic changes considering long-COVID and related outcomes; these aspects should be considered by future studies. External validation of our findings in independent cohorts will be important to confirm the robustness and generalizability of the DNAm changes identified, as this study only included European cohorts representative from the early pandemic.

## Conclusion

This study represents the first large-scale EWAS examining the long-term epigenetic landscape of mild SARS-CoV-2 infection. Our findings indicate moderate but persistent changes in whole blood up to 4 months post-infection. This study adds to the growing evidence on the long-term epigenetic signatures of COVID-19 infection and its overlap with inflammation, metabolic and immune dysregulation. We confirmed associations previously reported for *IFI44L*, *MX1*, *DDX60*, *PARP9* and *GNA12*, and identify additional regions warranting further research. This study identifies persistent epigenetic injuries acquired during acute phase of disease and presents initial evidence suggesting a few of these changes to be correlated with quality of life following an infection. Future longitudinal studies having concomitant DNAm and omics data should investigate the downstream effects of epigenetic changes and their change over time; the study setup of future studies should also consider appropriate collection of data on clinical symptoms long after an infection to contribute to molecular understanding of epigenetic changes associated with post-acute sequelae of SARS-CoV-2 infection.

## Supplementary Information

Supplementary Material 1

## Data Availability

NAKO: Data of the NAKO are generally not available to the public due to strict data protection regulations. However, scientists can apply for data use according to the official usage regulation specifications. Please refer to https://transfer.nako.de for further information.Lifelines: Data may be obtained from a third party and are not publicly available. Researchers can apply to use the Lifelines data used in this study. More information about how to request Lifelines data and the conditions of use can be found on their website.CON-VINCE: The dataset for this manuscript is not publicly available as it is linked to the CON-VINCE Study and its internal regulations. Any requests for accessing the dataset can be directed at con-vince@lih.lu. All data of the manuscript will be provided upon reasonable request and approval by the ethics committee.TiKoCo: The dataset for this manuscript is not publicly available as it is linked to the TiKOCo Study and its internal regulations. Any requests for accessing the dataset can be directed to the Study PIs, Prof. Dr. Ralf Wanger (ralf.wagner@ukr.de) and Prof. Dr. med. Klaus Überla (klaus.ueberla@uk-erlangen.de).DNAm datasets: DNAm datasets are stored independently by each cohort and therefore protected by the same data use and access policies mentioned above.
