# Supplementary material for: DNAm landscape up to 4 months post SARS-CoV-2 infection: insights from four population-based cohorts
Source: Clin Epigenetics. 2026 Jun 13;18:116. doi: 10.1186/s13148-026-02166-1 (PMC13263947; doi:10.1186/s13148-026-02166-1)
Supplement: Supplementary file 1 — Supplementary Material 1 [file 13148_2026_2166_MOESM1_ESM.zip › Orchestra_Manuscript-SupplFile_v20260507.docx]

**DNAm landscape up to 4 months post SARS-CoV-2 infection: insights from four population-based cohorts**

**Supplemental File Contents**

[Supplemental Figures 3](#_Toc229053906)

[Supplemental Figure 1. QC plots done on study-level results from NAKO 3](#_Toc229053907)

[Supplemental Figure 2. QC plots done on study-level results from CON-VINCE 4](#_Toc229053908)

[Supplemental Figure 3. QC plots done on study-level results from Lifelines 5](#_Toc229053909)

[Supplemental Figure 4. QC plots done on study-level results from TiKoCo 6](#_Toc229053910)

[Supplemental Figure 5. Effect sizes and precision by sample size from study-level EWAS results 7](#_Toc229053911)

[Supplemental Figure 6. Leave-one-out analyses for cg03607951 8](#_Toc229053912)

[Supplemental Figure 7. Leave-one-out analyses for cg24678928 8](#_Toc229053913)

[Supplemental Figure 8. GO Terms and KEGG Pathways represented in list of 117 CpGs 9](#_Toc229053914)

[Supplemental Figure 9. Gene set enrichment (WebGestalt) 10](#_Toc229053915)

[Supplemental Figure 10. Chromatin states represented in list of suggestive CpGs 10](#_Toc229053916)

[Supplemental Figure 11. Cis-eQTM plots for top CpGs from DMP and DMR analyses 11](#_Toc229053917)

[Supplemental Tables 12](#_Toc229053918)

[Supplemental Table 1. NAKO EWAS results 12](#_Toc229053919)

[Supplemental Table 2. CONVINCE EWAS results 12](#_Toc229053920)

[Supplemental Table 3. Lifelines EWAS results 12](#_Toc229053921)

[Supplemental Table 4. TiKoCo EWAS results 12](#_Toc229053922)

[Supplemental Table 5. Meta-analysis results from model adjusting for age, sex and WBCs 12](#_Toc229053923)

[Supplemental Table 6. Comparison of meta-analysis results between models 12](#_Toc229053924)

[Supplemental Table 7. Meta-analysis results from model additionally adjusting for smoking and chronic disease 12](#_Toc229053925)

[Supplemental Table 8. Serology-based sensitivity analysis 12](#_Toc229053926)

[Supplemental Table 9. Description of meta-DMRs 12](#_Toc229053927)

[Supplemental Table 10. Sensitivity DMR analyses 12](#_Toc229053928)

[Supplemental Table 11. Replication of meta-DMR across sensitivity analyses 12](#_Toc229053929)

[Supplemental Table 12. Gene-level overlap across DMR analyses 13](#_Toc229053930)

[Supplemental Table 13. Cohort-level DMR results 13](#_Toc229053931)

[Supplemental Table 14. Replication of meta-DMR across cohorts 13](#_Toc229053932)

[Supplemental Table 15. Gene-level overlap across meta- and cohort-DMRs 13](#_Toc229053933)

[Supplemental Table 16. Gene Ontology enrichment results 13](#_Toc229053934)

[Supplemental Table 17. KEGG Pathway enrichment results 13](#_Toc229053935)

[Supplemental Table 18. Gene expression in immune cells (HPA) 13](#_Toc229053936)

[Supplemental Table 19. Webgestalt Gene sets 13](#_Toc229053937)

[Supplemental Table 20. Chromatin states 13](#_Toc229053938)

[Supplemental Table 21. eQTM analyses 14](#_Toc229053939)

[Supplemental Table 22. Literature annotation 14](#_Toc229053940)

[Supplemental Notes 15](#_Toc229053941)

[Supplemental Note 1: Cohort-specific information 15](#_Toc229053942)

[Supplemental Note 2: DNA methylation preprocessing and QC 19](#_Toc229053943)

[Supplemental Note 3: KORA FF4 DNA methylation and expression data 22](#_Toc229053944)

[Group authorships 23](#_Toc229053945)

[References 28](#_Toc229053946)

# Supplemental Figures

## Supplemental Figure 1. QC plots done on study-level results from NAKO


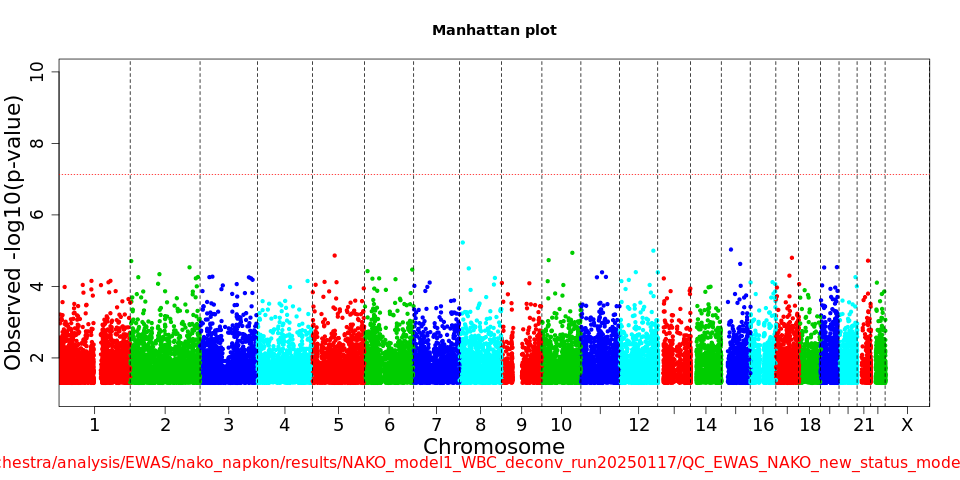

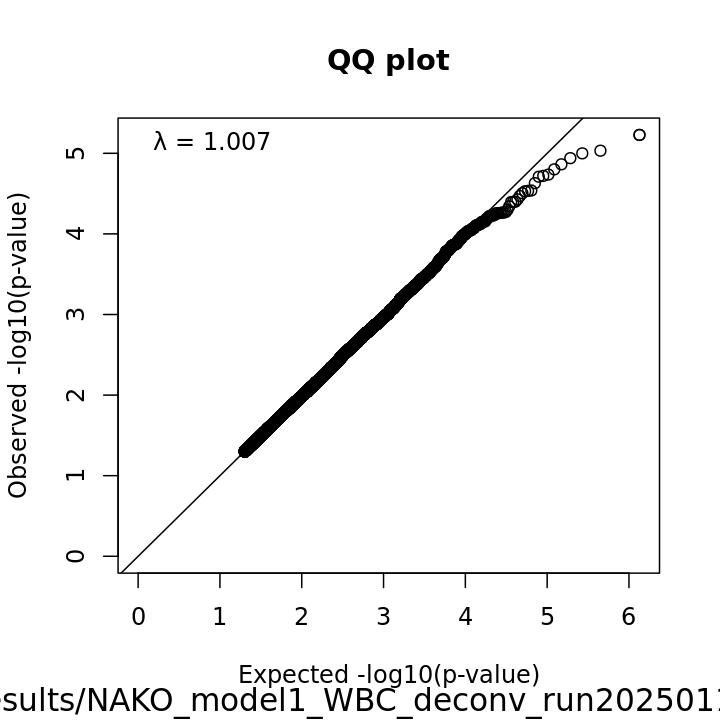

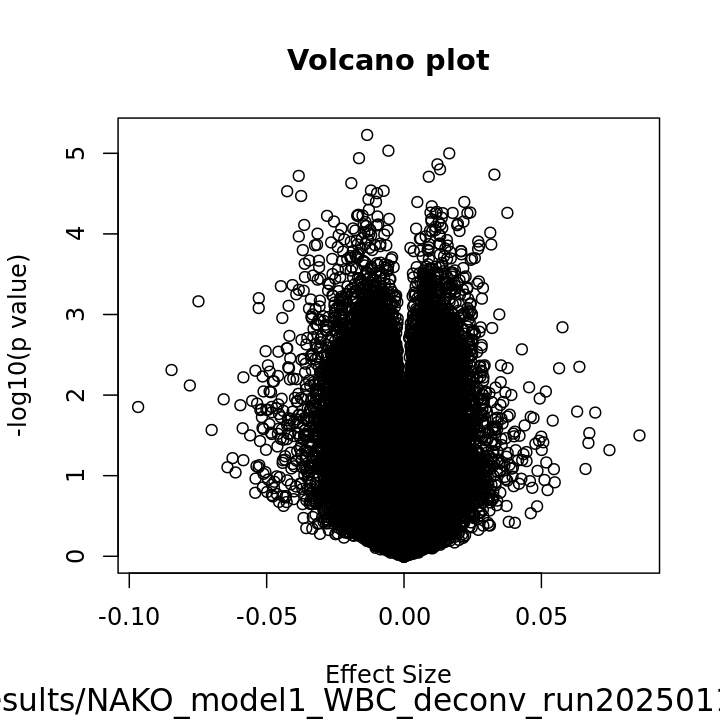

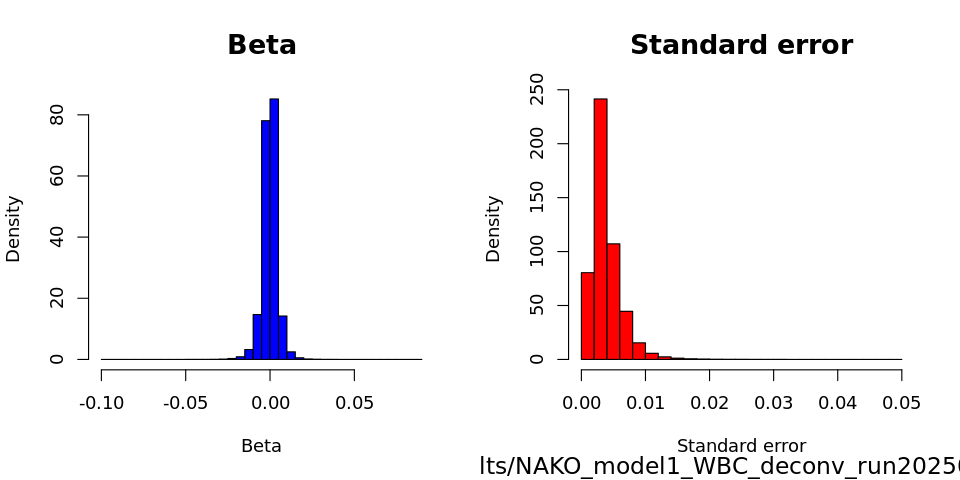


**A**

**B**

**C**

**D**


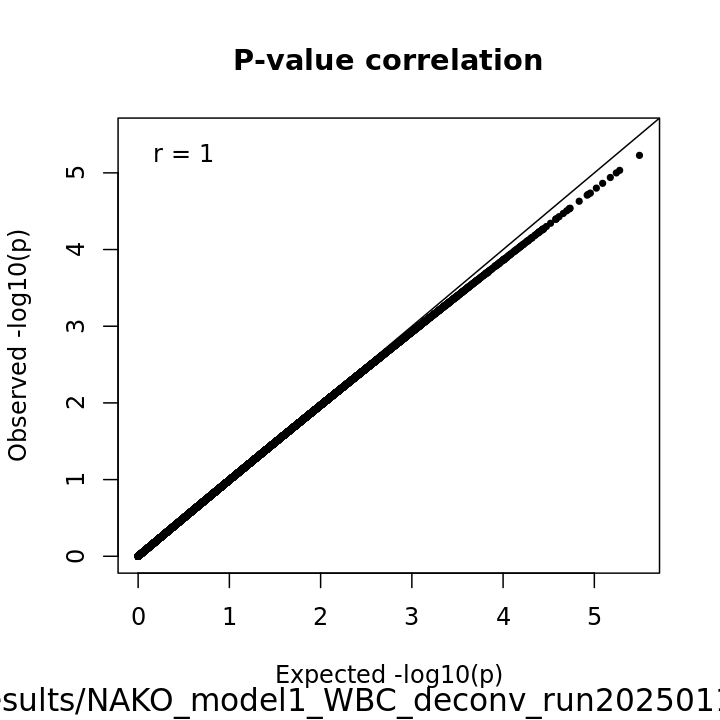


Figure Caption. A. Manhattan plot showing associations between genome-wide CpG probes and case status adjusted for age, sex, DNAm-based estimated cell proportions, smoking, chronic disease and EPIC chip version. The horizontal red line denotes the significance threshold based on the Bonferroni correction; points above this threshold are considered statistically significant. Each data point on the plot represents an epigenetic marker tested in the study, where they are colored based on the chromosome. The higher a point on the y-axis, the stronger the association with case status. B. Q-Q plot and p-value correlation scatterplot comparing observed vs expected -log10 p values. C. Histograms of beta coefficient and standard errors observed in EWAS. D. Volcano plot showing effect sizes in x-axis and statistical significance (-log10 pval) in y-axis.

## Supplemental Figure 2. QC plots done on study-level results from CON-VINCE


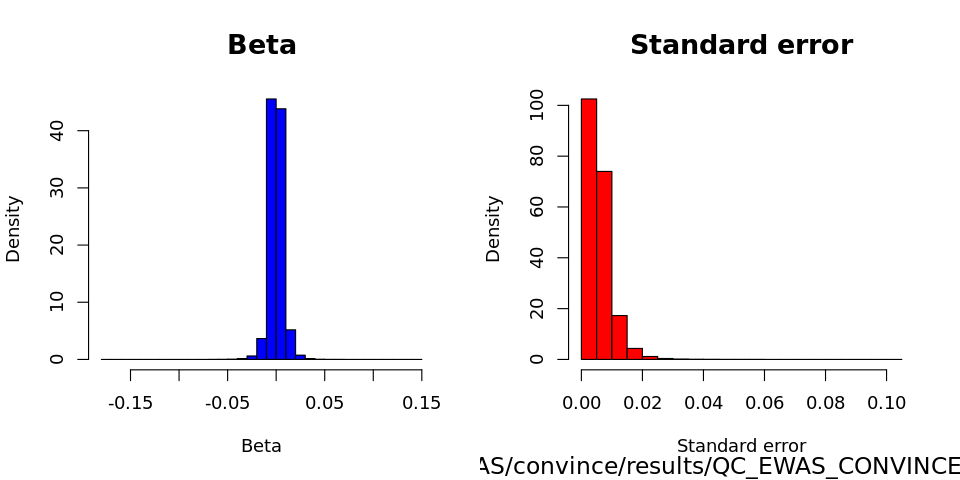

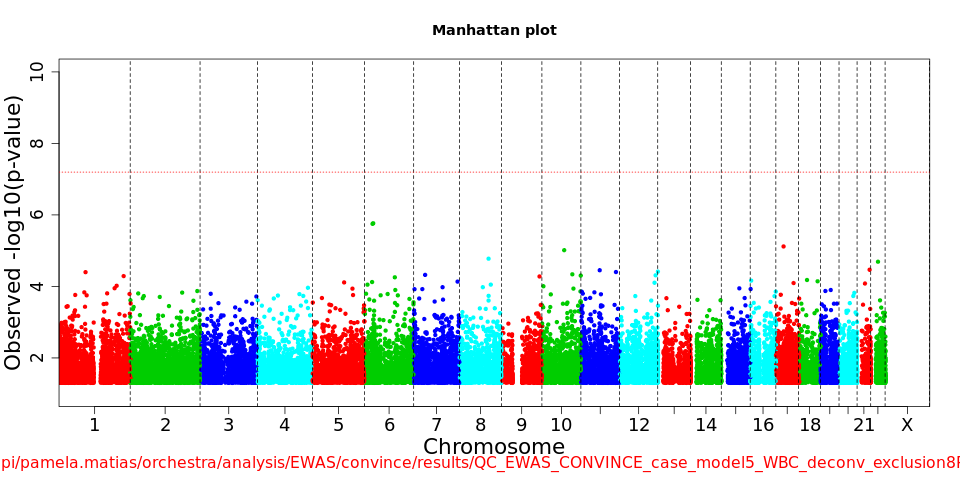

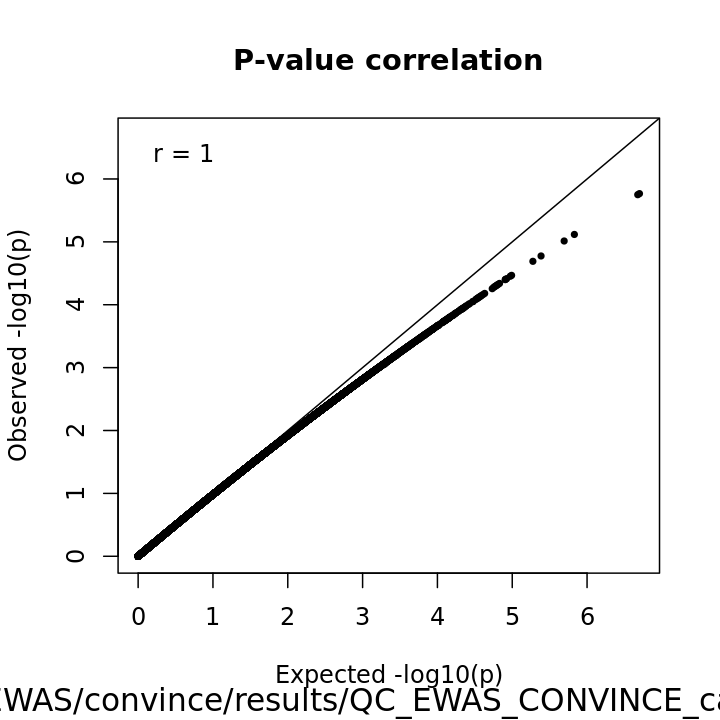

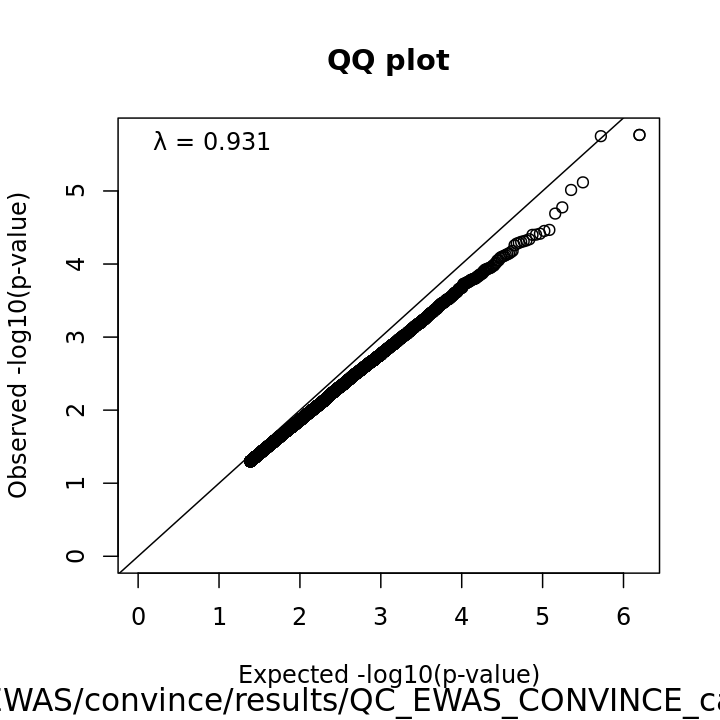

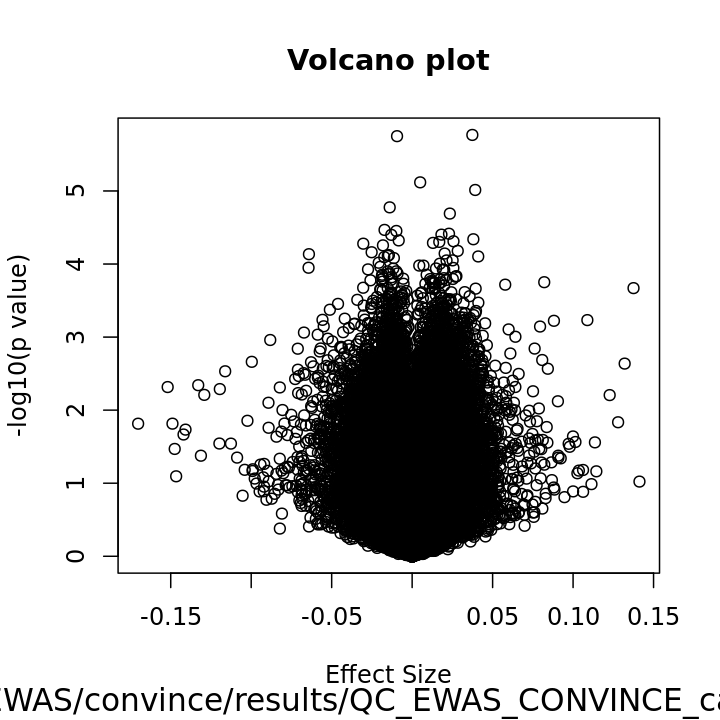


**A**

**B**

**C**

**D**

Figure Caption. A. Manhattan plot showing associations between genome-wide CpG probes and case status adjusted for age, sex, DNAm-based estimated cell proportions, smoking, and chronic disease. The horizontal red line denotes the significance threshold based on the Bonferroni correction; points above this threshold are considered statistically significant. Each data point on the plot represents an epigenetic marker tested in the study, where they are colored based on the chromosome. The higher a point on the y-axis, the stronger the association with case status. B. Q-Q plot and p-value correlation scatterplot comparing observed vs expected -log10 p values. C. Histograms of beta coefficient and standard errors observed in EWAS. D. Volcano plot showing effect sizes in x-axis and statistical significance (-log10 pval) in y-axis.

## Supplemental Figure 3. QC plots done on study-level results from Lifelines


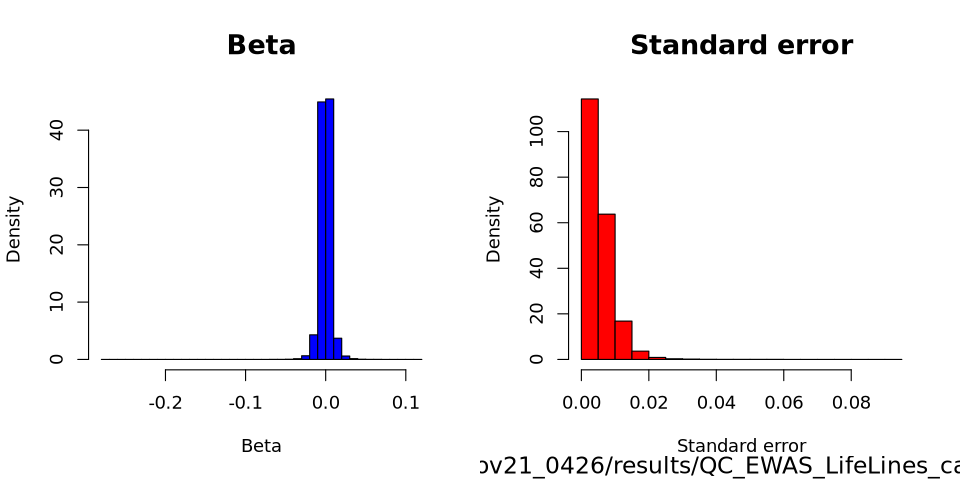

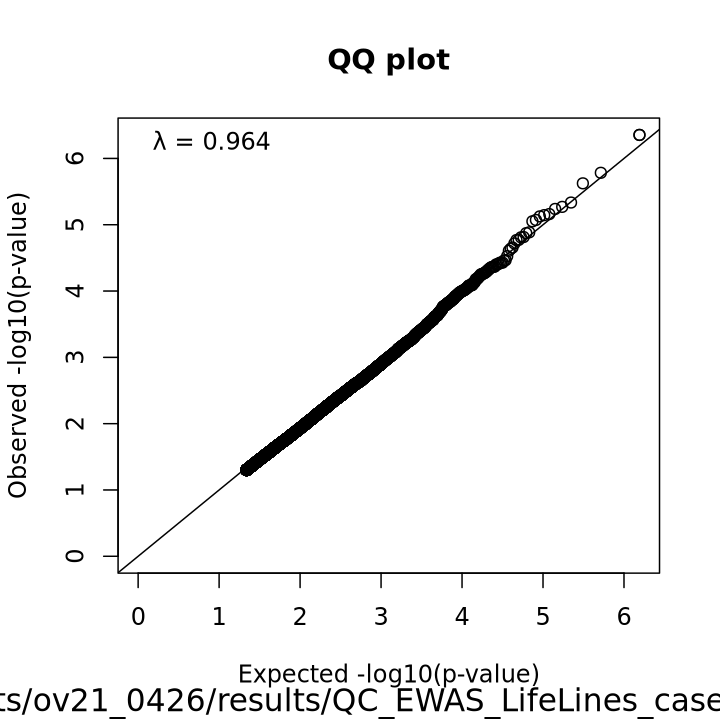

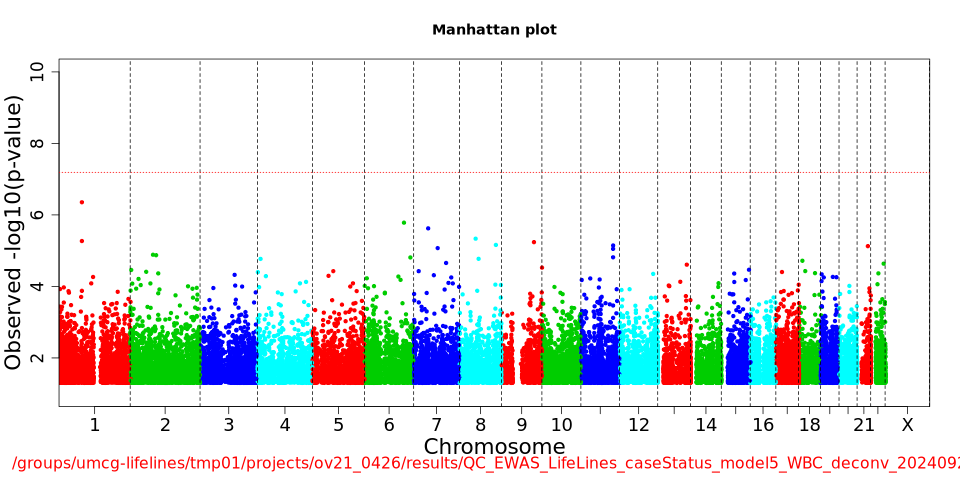


**A**


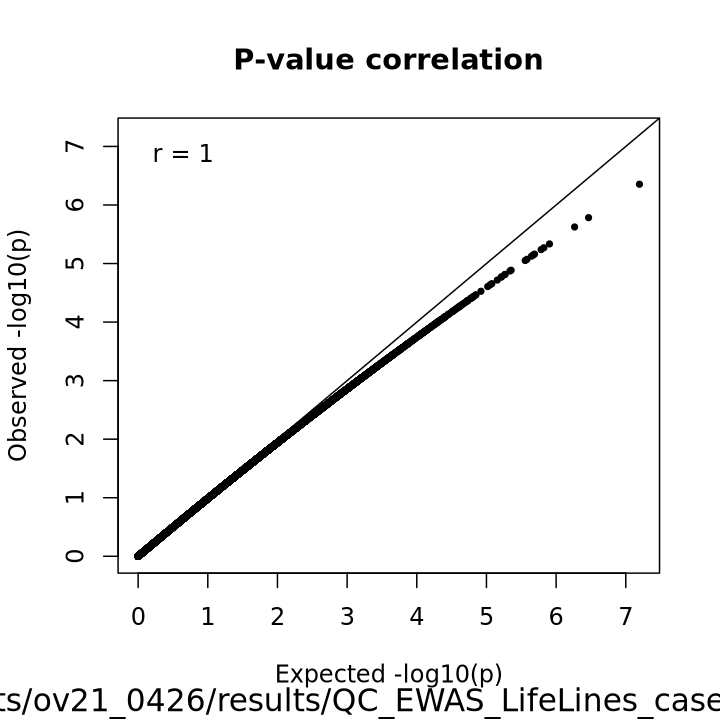


**B**

**C**


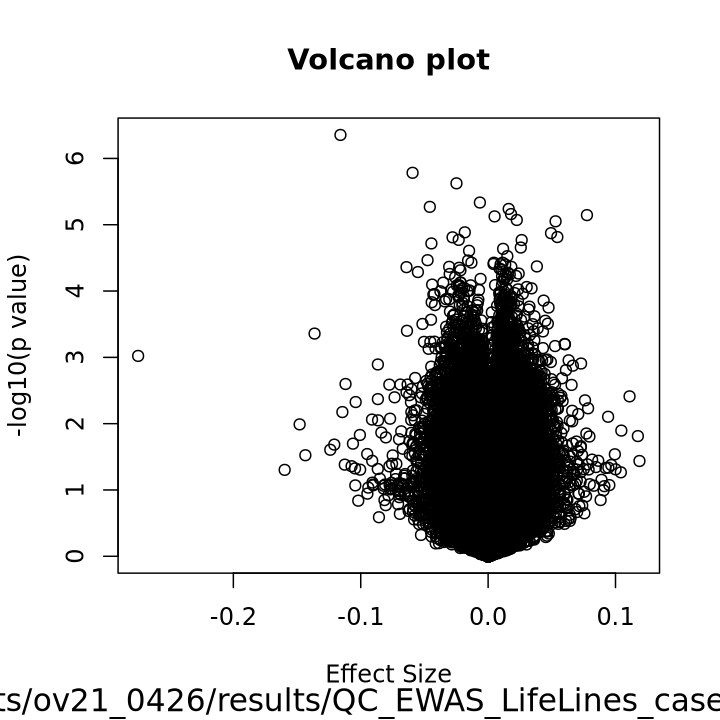


**D**

Figure Caption. A. Manhattan plot showing associations between genome-wide CpG probes and case status adjusted for age, sex, DNAm-based estimated cell proportions, smoking, and chronic disease. The horizontal red line denotes the significance threshold based on the Bonferroni correction; points above this threshold are considered statistically significant. Each data point on the plot represents an epigenetic marker tested in the study, where they are colored based on the chromosome. The higher a point on the y-axis, the stronger the association with case status. B. Q-Q plot and p-value correlation scatterplot comparing observed vs expected -log10 p values. C. Histograms of beta coefficient and standard errors observed in EWAS. D. Volcano plot showing effect sizes in x-axis and statistical significance (-log10 pval) in y-axis.

## Supplemental Figure 4. QC plots done on study-level results from TiKoCo


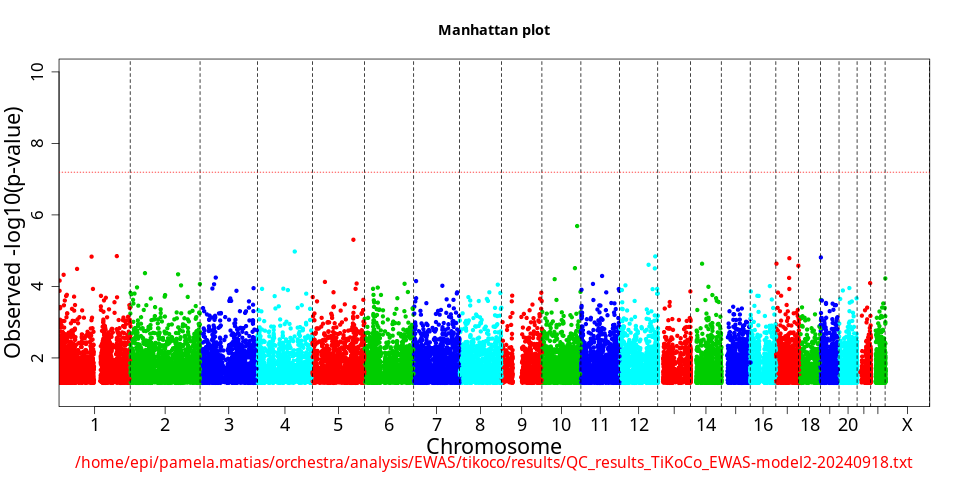

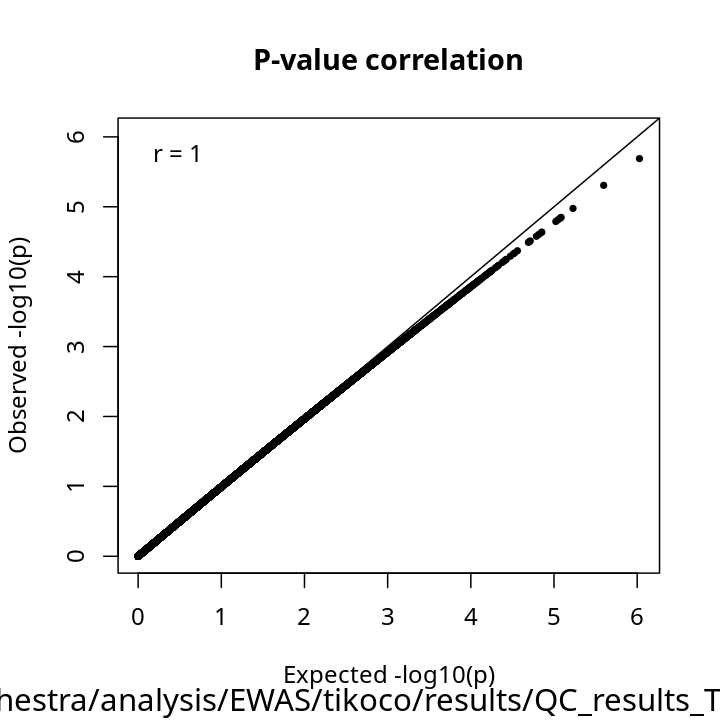

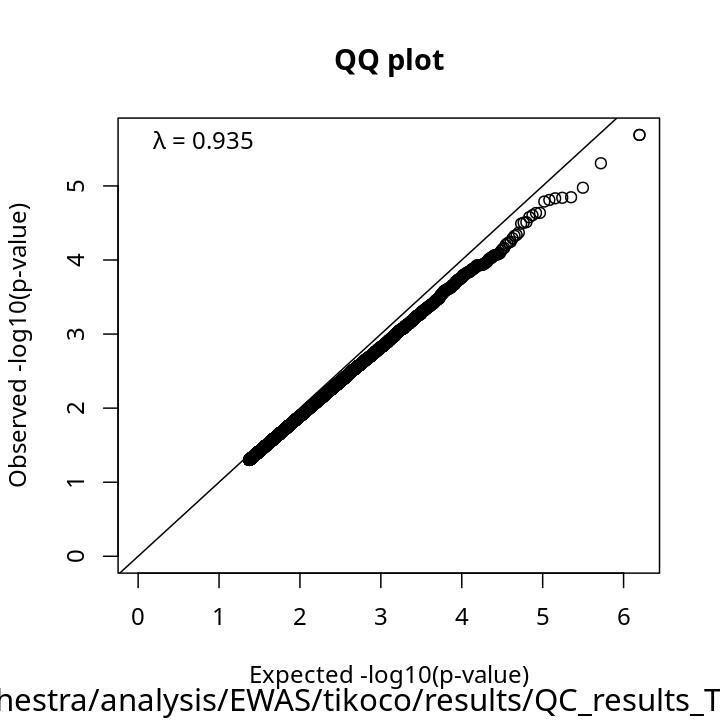

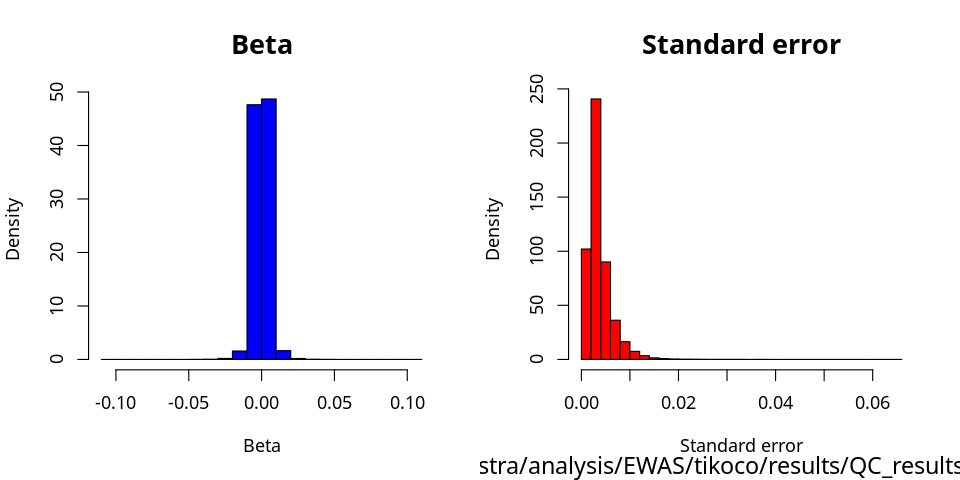

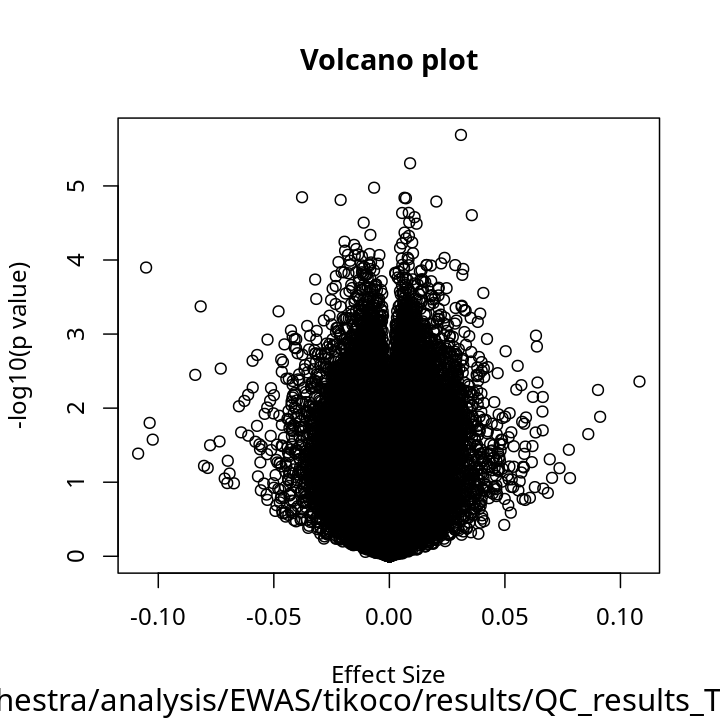


**C**

**D**

**A**

**B**

Figure Caption. A. Manhattan plot showing associations between genome-wide CpG probes and case status adjusted for age, sex, DNAm-based estimated cell proportions, smoking, and chronic disease. The horizontal red line denotes the significance threshold based on the Bonferroni correction; points above this threshold are considered statistically significant. Each data point on the plot represents an epigenetic marker tested in the study, where they are colored based on the chromosome. The higher a point on the y-axis, the stronger the association with case status. B. Q-Q plot and p-value correlation scatterplot comparing observed vs expected -log10 p values. C. Histograms of beta coefficient and standard errors observed in EWAS. D. Volcano plot showing effect sizes in x-axis and statistical significance (-log10 pval) in y-axis.

## Supplemental Figure 5. Effect sizes and precision by sample size from study-level EWAS results


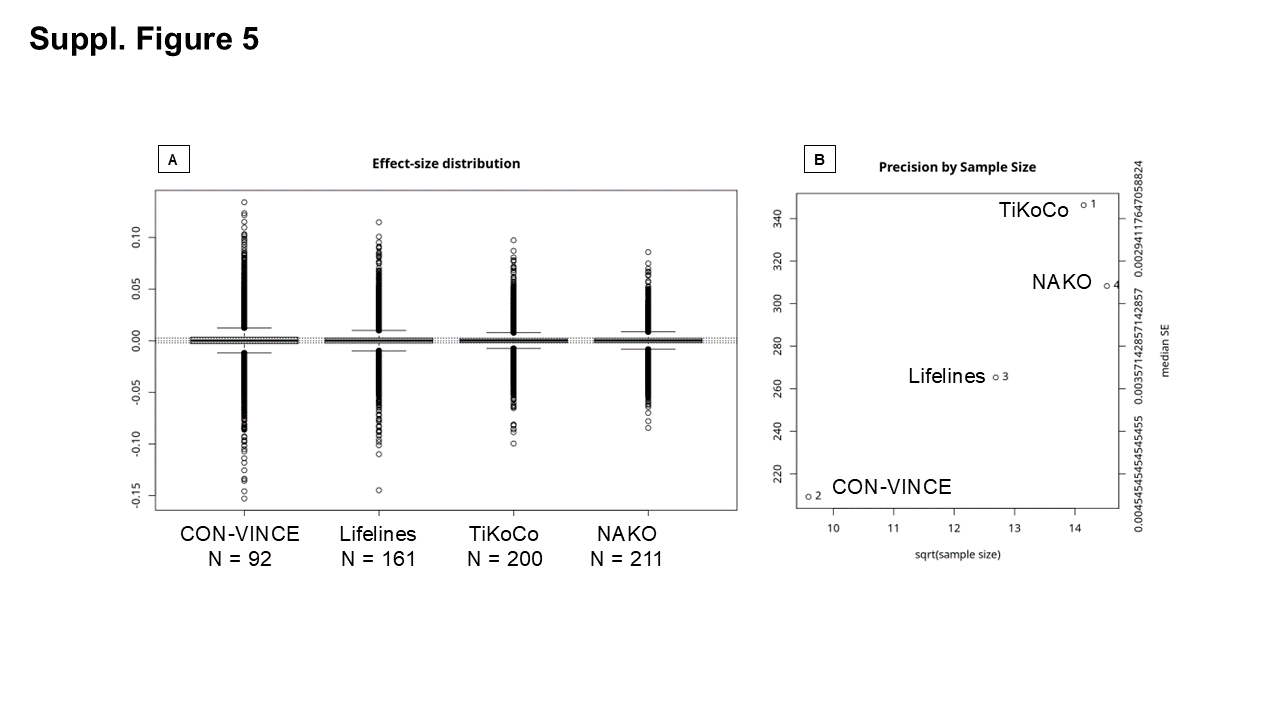


**Caption Suppl. Figure 5.**

Plots showing distribution of effect sizes across study-level EWAS results (panel A) and precision of study-level EWAS estimates plotted against square root of sample size (panel B), where higher precision is expected in larger cohorts.

##
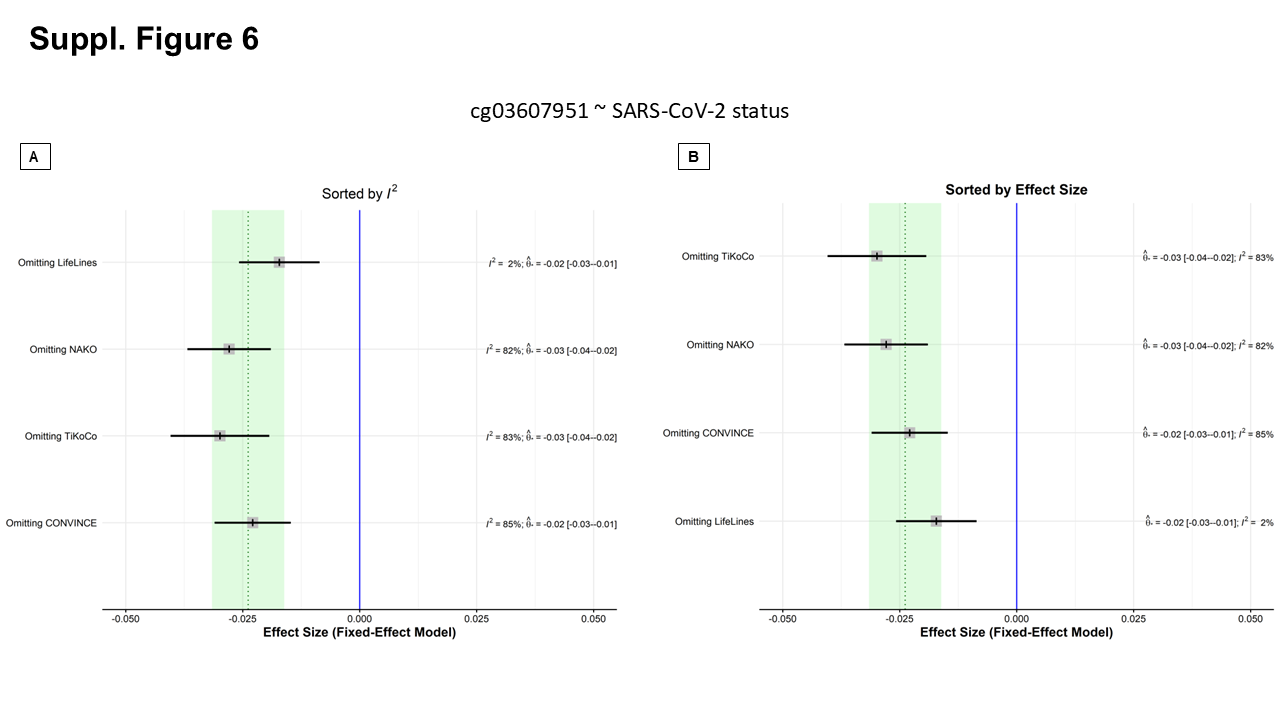
Supplemental Figure 6. Leave-one-out analyses for cg03607951

## Supplemental Figure 7. Leave-one-out analyses for cg24678928


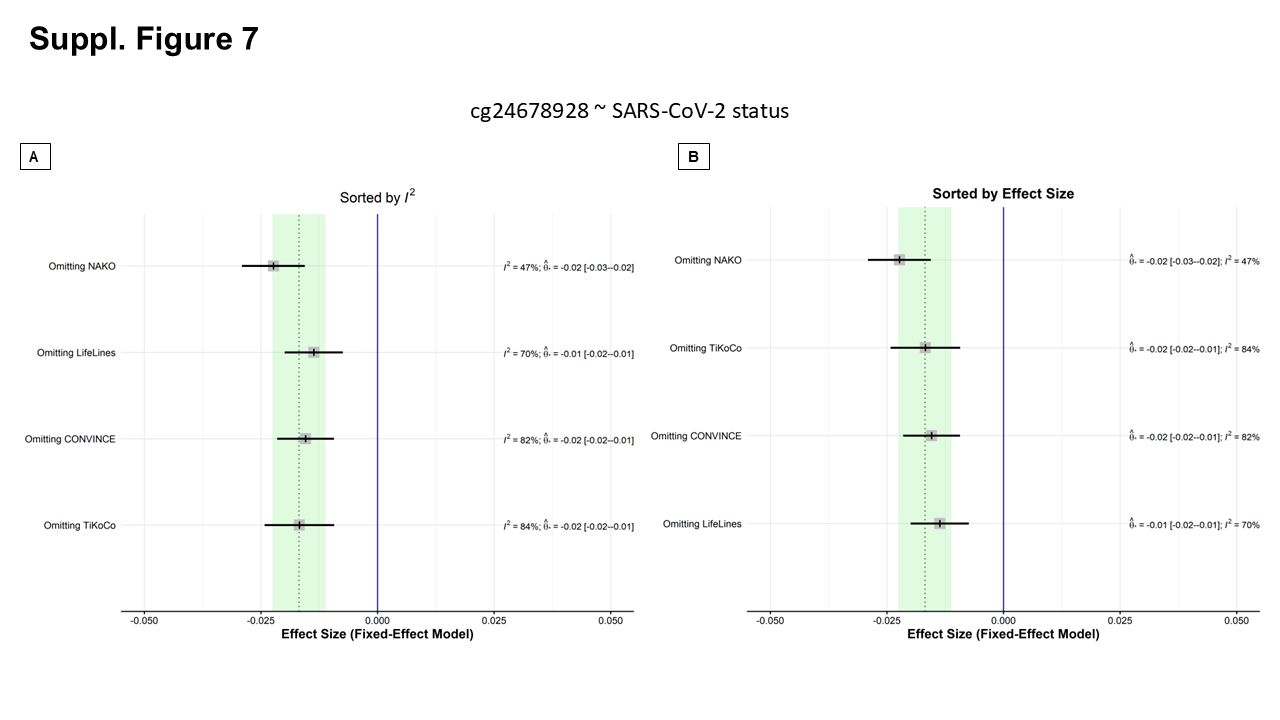


**Caption Suppl. Figures 6 and 7.**

Forest plots showing pooled effects and I^2^ heterogeneity of all fixed-effects meta-analyses done leaving one study out each time. Dashed grey line and area shaded with green represent the estimated pooled effect and its 95% confidence interval. Plot sorted by the pooled effect size (panel A) and by the I2 value of the leave-one-out meta-analyses (panel B). Shown for each meta-analysis are also the estimated pooled effects (θ) with their corresponding 95% CI and I^2^ value.

## Supplemental Figure 8. GO Terms and KEGG Pathways represented in list of 117 CpGs

1.
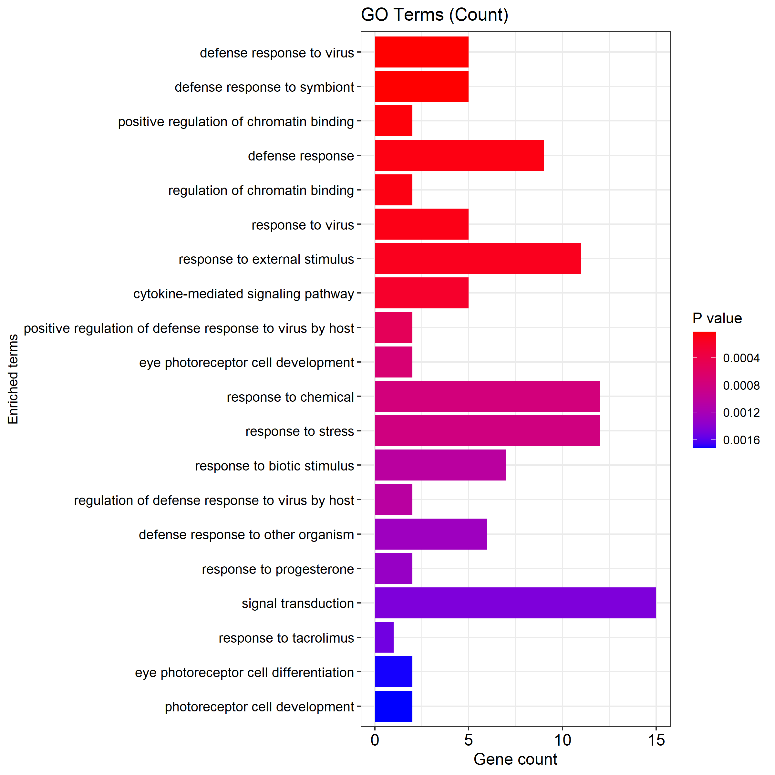

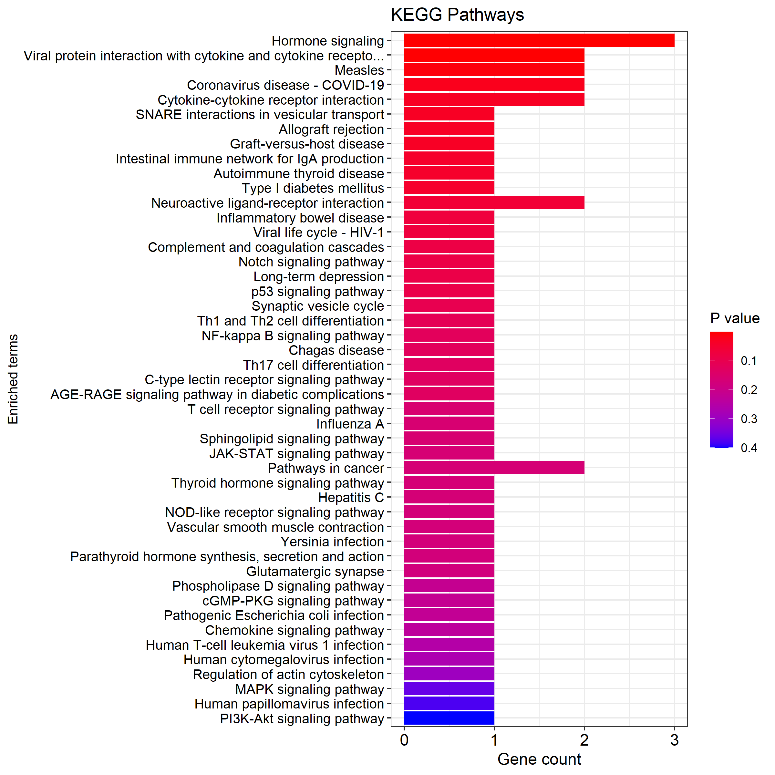
 B)

A. Barplot showing GO terms (y-axis) and the gene count (x-axis) as estimated in GO enrichment analyses. B. Barplot showing GO terms (y-axis) and the gene count (x-axis) as estimated in KEGG pathway enrichment analyses.

## Supplemental Figure 9. Gene set enrichment (WebGestalt)


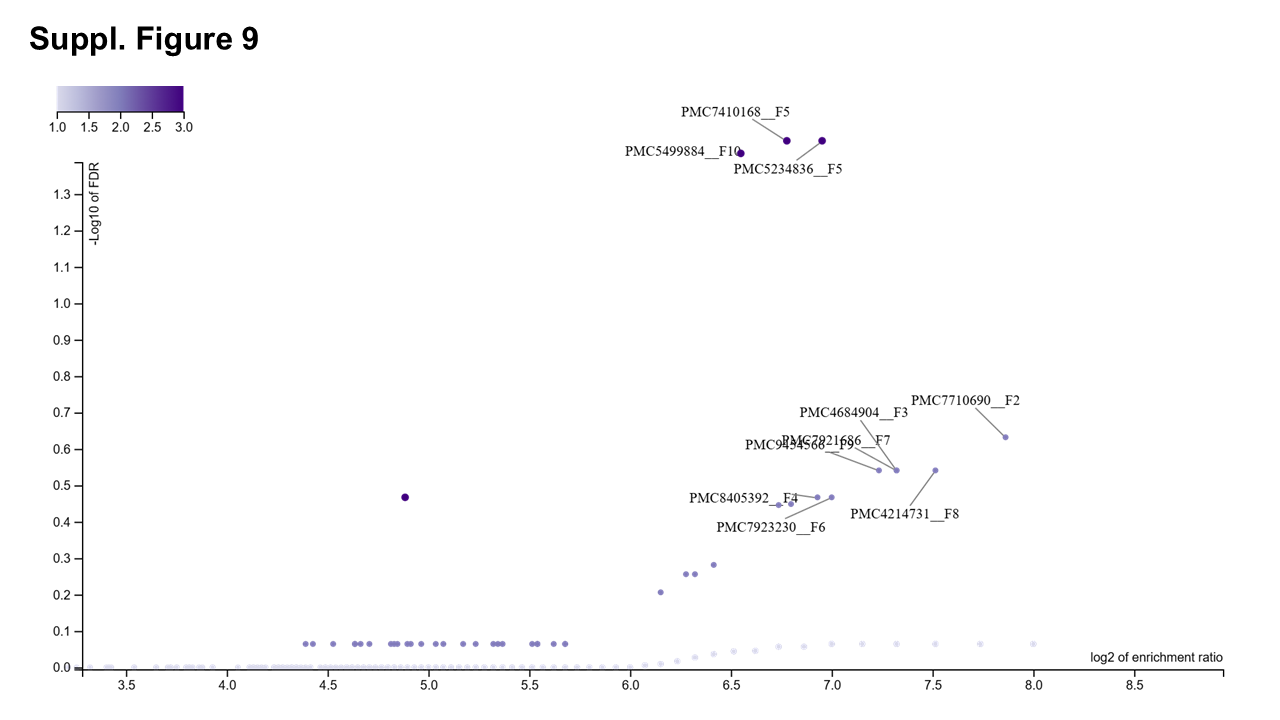


Caption. Gene sets enriched as identified in overrepresentation analyses conducted using Webgestalt (1). Y-axis shows -log10 of FDR from enrichment analysis, x-axis shows log2 values of enrichment ratio between genes represented in query and genes represented in gene sets.

## Supplemental Figure 10. Chromatin states represented in list of suggestive CpGs


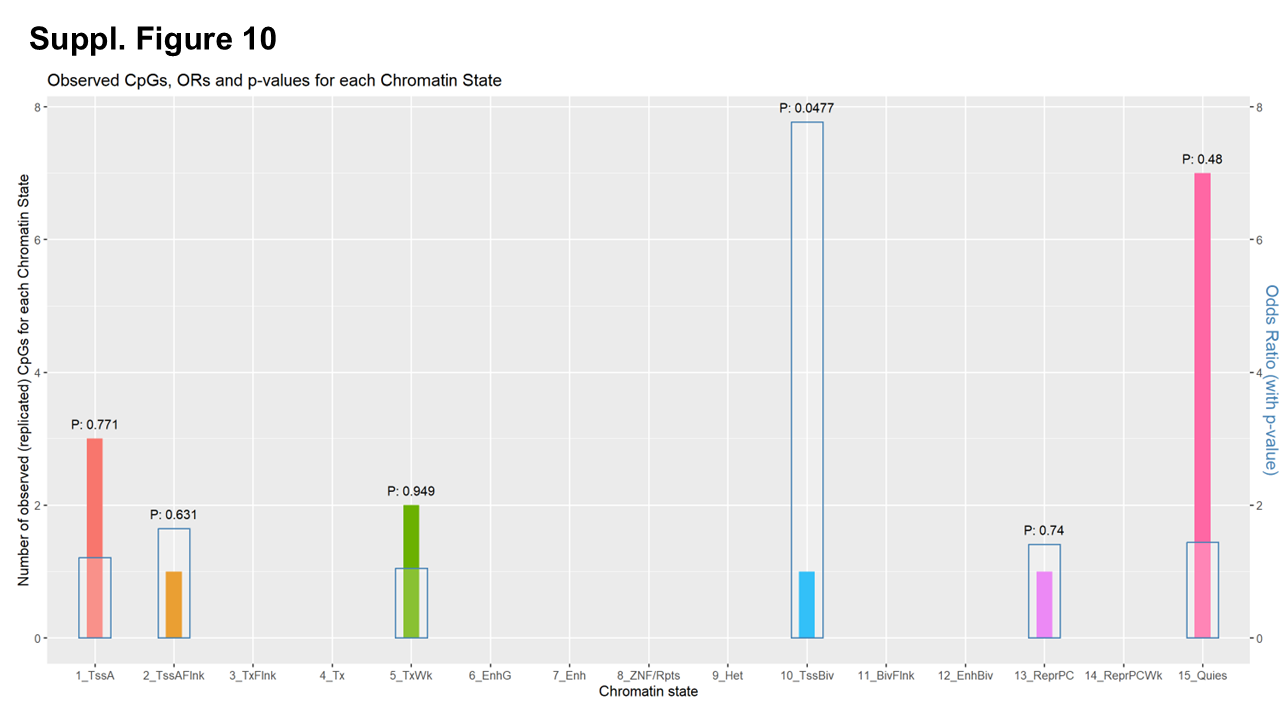


Caption. X-axis shows 15 chromatin states, y-axis displays number of observed CpGs in each chromatin state (left) and odds ratio resulting from enrichment analysis (right). Colored bars represent the number of CpGs found for each chromatin state, the transparent bars represent the ORs and p-values obtained are shown on top of the bars.

## Supplemental Figure 11. Cis-eQTM plots for top CpGs from DMP and DMR analyses


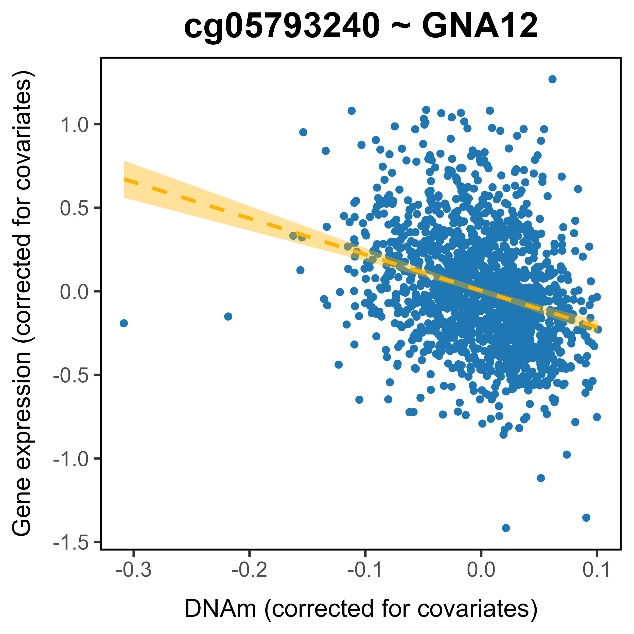

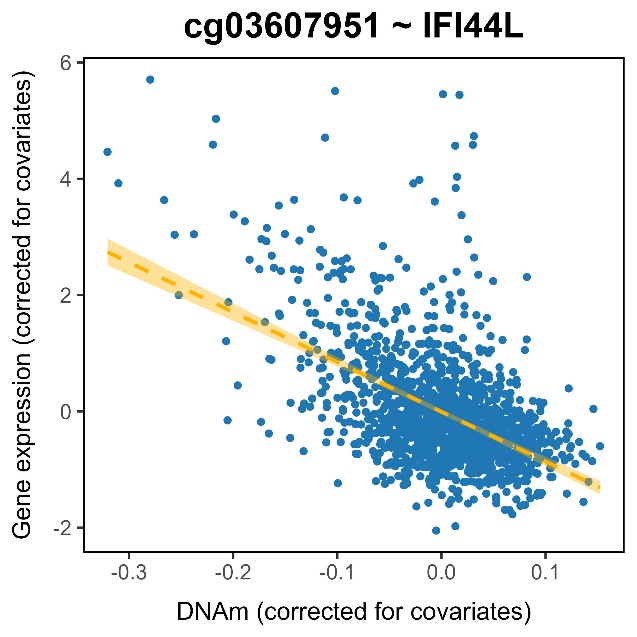


A) B)


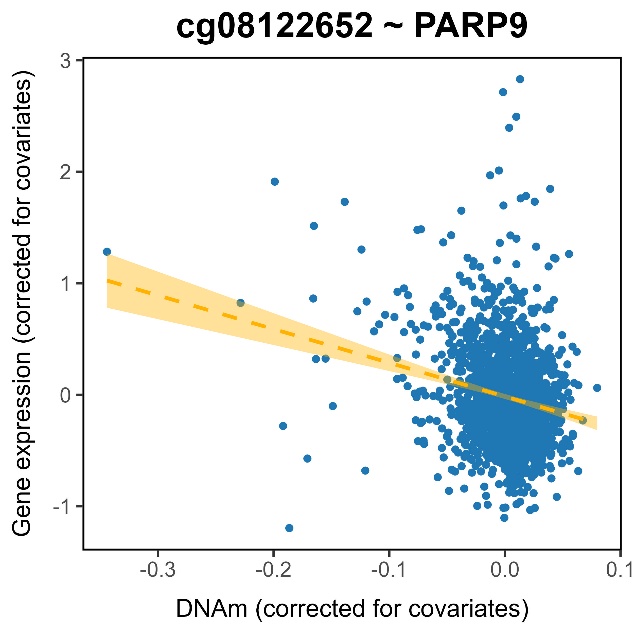


C)

Selected cis‑eQTM results linking CpG methylation to nearby gene expression in the KORA FF4 cohort (n = 1,543). eQTM analyses were performed using MatrixEQTL on DNAm and RNA‑seq data, testing transcripts within 500 kb of each CpG and adjusting for age, sex, white blood cell proportions, and technical factors; significance was determined using Bonferroni correction. Panels show robust negative associations for A) cg03607951 with *IFI44L*, B) cg05793240 (most significant CpG in DMR4) with *GNA12* and C) cg08122652 (most significant CpG in DMR10) with *PARP9*. Each panel shows DNAm residuals (x‑axis) plotted against gene expression residuals (y‑axis), with fitted regression lines and 95% confidence intervals.

# Supplemental Tables

## Supplemental Table 1. NAKO EWAS results

EWAS in NAKO, CpG ~ case status + age + sex + WBCs + ChipVersion

## Supplemental Table 2. CONVINCE EWAS results

EWAS in CON-VINCE, CpG ~ case status + age + sex + WBCs

## Supplemental Table 3. Lifelines EWAS results

EWAS in Lifelines, CpG ~ case status + age + sex + WBCs

## Supplemental Table 4. TiKoCo EWAS results

EWAS in TiKoCo, CpG ~ case status + age + sex + WBCs

## Supplemental Table 5. Meta-analysis results from model adjusting for age, sex and WBCs

Meta-EWAS from NAKO, CON-VINCE, Lifelines, TiKoCo: CpG ~ case status + age + sex + WBCs

## Supplemental Table 6. Comparison of meta-analysis results between models

Comparison between main EWAS model adjusting for age, sex and WBCs to model additionally adjusting for chronic disease and smoking for 16 CpGs p < 1e-05 from main model

## Supplemental Table 7. Meta-analysis results from model additionally adjusting for smoking and chronic disease

Meta-EWAS from NAKO, CON-VINCE, Lifelines, TiKoCo: CpG ~ case status + age + sex + smoking + chronic disease

## Supplemental Table 8. Serology-based sensitivity analysis

Results from sensitivity analysis done with participant data from cohorts with serology-based case definition (TiKoCo and CONVINCE, N = 292)

## Supplemental Table 9. Description of meta-DMRs

Summary of differentially methylated regions (DMRs), including genomic coordinates, Sidak‑adjusted significance, number of probes per region, overlap with study‑level DMPs, distribution of DMP p‑values, mean effect size, direction concordance across probes, and annotated genes.

## Supplemental Table 10. Sensitivity DMR analyses

DMRs detected under four alternative DMR‑calling parameter settings (SA1–SA4), which differ in window size (d), bandwidth (b), and smoothing threshold (s). The tables report region‑level significance, probe counts, and the specific CpGs included in each DMR, enabling comparison of DMR robustness across parameter choices.

## Supplemental Table 11. Replication of meta-DMR across sensitivity analyses

Comparison of DMRs identified in the main analysis with results from the four sensitivity analyses (SA1–SA4). For each DMR, the table reports genomic coordinates, region‑level significance (p, FDR, Sidak), number of contributing probes, CpG list, and annotated genes. Columns indicate whether the region was also detected in each sensitivity analysis (rep_region_SA1–SA4), the number of overlapping CpGs (cpg_overlap_SA1–SA4), and the number of overlapping annotated genes (gene_overlap_SA1–SA4).

## Supplemental Table 12. Gene-level overlap across DMR analyses

Gene‑level overlap between meta‑DMRs and DMRs identified in each DMR analysis (main, SA1 through SA4). For each gene, the table indicates whether it was detected in the meta‑analysis and whether it appeared in the DMR results of each sensitivity analysis. This summary highlights genes consistently replicated across cohorts as well as those unique to individual datasets.

## Supplemental Table 13. Cohort-level DMR results

Overview of DMRs identified in the meta‑analysis and in each of the four cohorts (Lifelines, TiKoCo, NAKO, CONVINCE). For each DMR, the table reports a cohort-specific ID, genomic coordinates, statistical significance, number of contributing CpGs, and the full CpG list. CpG‑level annotations (gene assignment, regulatory feature, CpG island context, and nearby SNPs) are provided.

## Supplemental Table 14. Replication of meta-DMR across cohorts

Comparison of meta‑DMRs with cohort‑specific DMRs from TiKoCo, Lifelines, NAKO, and CONVINCE. For each meta‑DMR, the table reports genomic coordinates, region‑level significance (p, FDR, Sidak), number of CpGs, and annotated genes. Replication indicators (rep_region_c1–c4) denote whether the corresponding region was detected in each cohort. Overlap metrics (cpg_overlap and gene_overlap) quantify the number of CpGs and genes shared between the meta‑DMR and each cohort‑level DMR.

## Supplemental Table 15. Gene-level overlap across meta- and cohort-DMRs

Comparison of gene‑level findings across the meta‑DMR analysis and four cohort‑specific DMR analyses. TRUE/FALSE indicators show whether each gene was detected in the meta‑analysis and in TiKoCo, Lifelines, NAKO, and CONVINCE.

## Supplemental Table 16. Gene Ontology enrichment results

GO enrichment full output of pathways represented in set of 117 CpGs (from either DMP or DMR analyses) with more than one gene (DE >1)

## Supplemental Table 17. KEGG Pathway enrichment results

KEGG Pathways represented in set of 16 CpGs associated with case status at p < 1e-05 with more than one gene represented in pathway (DE >1)

## Supplemental Table 18. Gene expression in immune cells (HPA)

Integrated CpG‑level annotation for DMPs and meta‑DMRs, including chromosome, position, gene annotation, and immune‑cell expression specificity derived from the Human Protein Atlas (HPA). The HPA immune‑cell expression resource is based on single‑cell RNA‑seq across 18 immune cell types, allowing genes to be categorized by specificity (e.g., low, moderate, or enriched in particular immune subsets). Classification (e.g., low immune‑cell specificity) and URLs of the corresponding HPA single‑cell expression pages are provided in the last two columns.

## Supplemental Table 19. Webgestalt Gene sets

WebGestalt Gene sets enriched in set of 16 CpGs associated with case status at p < 1e-05

## Supplemental Table 20. Chromatin states

Chromatin states represented in set of 16 CpGs associated with case status at p < 1e-05

## Supplemental Table 21. eQTM analyses

Overview of cis‑eQTM associations for CpGs identified in the DMP analysis and within each DMR. For each CpG, the table reports the corresponding gene transcript, FDR‑adjusted p‑value, and effect size.

## Supplemental Table 22. Literature annotation

Known roles and associations for genes annotated to significant DMP/DMRs.

# Supplemental Notes

## Supplemental Note 1: Cohort-specific information

#### **CON-VINCE**

##### Cohort description

CON-VINCE (COvid-19 National survey for assessing VIral spread by Non-affected CarriErs) was launched in April 2020 by Research Luxembourg COVID-19 task force to identify asymptomatic and mildly symptomatic individuals and follow them up for a year. It is a longitudinal observational study collecting self-reported survey information via online tools and biosamples via local private laboratories. CON-VINCE participants completed a survey and donated biological material bi-weekly, from April 2020 to June 2020 (Visits 0–4) and at the annual follow-up visit 6. One-three months before the annual visit, only online survey information was collected (Visit 5). Participants were tested for SARS-CoV-2 by RT-qPCR and anti-SARS-CoV-2 serology at all sampling visits. For additional details, please refer to (2).

##### Variable definition

- Chronic disease: prevalent cardiovascular, metabolic and/or lung disease as per the variable definitions described next.
- Cardiovascular disease: variable included in analysis defined by history of *cardiac infarction, angina pectoris and presence of stents*. Variable show in table 1 additionally including hypertension, defined as chronic elevated blood pressure over 140/90 or treatment by antihypertensive medication.
- Metabolic disease: self-reported prevalent diabetes.
- Lung disease: self-reported chronic pulmonary disease (chronic inflammatory lung disease- e.g. chronic obstructive pulmonary disease but without including asthma).
- Chronic disease variable shown in Table 1 additionally considers information on prevalent diabetes complications, rheumatologic disorders, liver disease, chronic neurological disorders (dementia).
- Smoking: Current smoking habits classified as never smokers (including individuals living with smokers), former smoker (including individuals who reported smoking after beginning of the pandemic in March 2020), and current smokers.
- Case definition: Participants were identified as infected in a case of a positive RT-qPCR or/and presence of IgG-S to SARS-CoV-2 prior to vaccination or/and presence of IgG-N to SARS-CoV-2 or/and self-reported positive SARS-CoV-2 test result (RT-qPCR in > 95% cases; other tests < 5% cases) observed at least at one of the visits.
- Cases and controls were matched following a “two-stage exact matching” strategy. In the first stage, matching is done based on sex (male/female), 5-year age groups and “nationality / country of origin”; this strategy produced 23 pairs. In the second stage, which is only for samples that are unmatched in the first stage, matching is done without “nationality / country of origin”; 27 pairs were identified in this second stage. A total of 50 pairs (100 samples) was identified in this cohort.
- Individuals with autoimmune disease (N=6; e.g. autoimmune thyroiditis-Hashimoto disease, Sjögren syndrome, Lupus erythematosus) and blood diseases (N=2; e.g. lymphoma, leukemia, multiple myeloma) were excluded from analysis.
- Epigenome-wide analysis of methylation sites was conducted using linear models with DNAm as outcome and case status as exposure, adjusting for age, sex, estimated white blood cell proportions (3): CpG ~ age + sex + 11 deconvoluted WBC proportions

#### **Lifelines**

##### Cohort description

Lifelines is a multi-disciplinary prospective population-based cohort study examining in a unique three-generation design the health and health-related behaviours of 167,729 persons living in the North of the Netherlands. It employs a broad range of investigative procedures in assessing the biomedical, socio-demographic, behavioural, physical and psychological factors which contribute to the health and disease of the general population, with a special focus on multi-morbidity and complex genetics (4, 5). Between 2006 and 2013, inhabitants of the northern part of The Netherlands and their families were invited to participate, thereby contributing to a three-generation design. Participants visited one of the Lifelines research sites for a physical examination, including lung function, ECG and cognition tests, and completed extensive questionnaires. Baseline data were collected for 167 729 participants, aged from 6 months to 93 years. Follow-up visits are scheduled every 5 years, and in between participants receive follow-up questionnaires. Linkage is being established with medical registries and environmental data. Lifelines contains information on biochemistry, medical history, psychosocial characteristics, lifestyle and more. (6). The Lifelines COVID-19 cohort is a subcohort of the Lifelines cohort that collected data about COVID-19–related symptoms, current health issues, and societal impacts from participants recruited from the Lifelines biobank. During the COVID-19 pandemic, participants from Lifelines received a series of COVID-19 questionnaires, and the ones who responded to ≥ 1 of the COVID-19 questionnaires sent were identified as participants of the Lifelines COVID-19 cohort. The Lifelines COVID-19 cohort has been partially validated against the COVID-19 infection status in northern Netherlands. COVID-19 questionnaires were sent on a (bi)weekly basis starting in March 2020 and on a monthly basis starting July 2020. In total, 22 questionnaires were sent until the end of July 2021. For additional information, refer to (7).

##### Variable definition and EWAS model

- Chronic disease: self-reported chronic health condition, including cardiovascular disease, high blood pressure, stroke, lung disease, liver disease, diabetes) collected in COVID questionnaire
- Case definition: Self-reported positive test within the 4 months prior to visiting the study center.
- Control definition: Controls were excluded if they tested positive preceding their visit to Lifelines, if they skipped the questions on testing status, fatigue, loss of taste and smell and coughing, or if they suffered from loss of taste and smell, fatigue or coughing, between the start of data collection but preceding their visit to Lifelines.
- Cases and controls were frequency-matched based on sex, age and month of visit to the study center.
- Epigenome-wide analysis of methylation sites was conducted using linear models with DNAm as outcome and case status as exposure, adjusting for age, sex, estimated white blood cell proportions (3):
- CpG ~ age + sex + 11 deconvoluted WBC proportions
- Smoking status: information on smoking habits collected in second questionnaire from second visit in third general assessment in Lifelines (3A Questionnaire 2). Categorized in never-smoker, current smoker, ex-smoker, and recent starter: “A person that smokes or has smoked for less than a year, is not seen as a smoker. Thus, a person that has smoked for a few months and then stopped is categorized as a 'never-smoker'. A person that recently started smoking and is currently still smoking is still seen as a never smoker because that person currently smokes for less then a year. However, these persons are categorized as a 'recent starter'.” Source: [smoking_tobacco_use [Lifelines Wiki]](https://wiki.lifelines.nl/doku.php?id=smoking_tobacco_use)

#### **NAKO**

##### Cohort description

The German National Cohort (NAKO) is a large prospective cohort study with 205,000 participants conducted in 18 study centers distributed all over Germany (8). Baseline examinations were conducted between 2014 and 2019, in which 205,415 individuals between 20–69 years old randomly selected from registration offices were examined. The second follow-up examination, planned for 5 years after the baseline examination, was finalized in 2024. A third follow-up examination is currently ongoing.

Between May and July 2020, the first year of the pandemic, all NAKO participants were re-contacted and a first supplementary COVID-19 questionnaire on SARS-CoV-2 infections and pandemic-related topics was sent out via email or letter to all study participants between April 30th and June 30th 2020. Overall, 160,227 questionnaires were completed, resulting in a response of 80.6% (9). NAKO conducted a second online survey between September and December 2022 focusing on SARS-CoV-2 infections and symptoms; 150,722 participants with valid email addresses were invited to participate in this survey, of which 110,375 (73.2%) completed it (10, 11).

##### Variable definition and EWAS model

- Chronic disease: prevalent chronic pulmonary disease or metabolic or cardiovascular disease at baseline, as defined in the next lines.
- Cardiovascular disease at baseline: self-reported heart infarction, angina pectoris, heart insufficiency, Herzrhythmusstörungen, hypertension or taking anti-hypertensive medication (ATC-Codes C02AC, C02CA, C02DB, C02DC, C03A, C03B, C03CA, C03CB, C03D, C03E, C07, C08CA, C08DA, C08DB, C08G, C09)
- Metabolic disease at baseline (self-reported diabetes at baseline survey, high blood lipids)
- Lung disease (self-reported COPD or chronic bronchitis at baseline survey)
- Smoking (self-reported at baseline)
- Case definition: Baseline NAKO examination completed, second examination done between 01.07.2020 and 30.06.2021, answered first COVID-19 questionnaire fully, were not vaccinated at the time of attending the second examination, had at least one test done at a doctor’s office, testing center or hospital since February 1 2020, at least one of these tests was positive and examination/blood draw was done within 0 to 130 days after the date of the positive COVID-19 test
- Control definition: Baseline NAKO examination completed, not vaccinated at the time of second examination, provided blood sample at second examination, never reported a COVID-19 infection
- Cases and controls were frequency-matched based on sex and age.
- Epigenome-wide analysis of methylation sites was conducted using linear models with DNAm as outcome and case status as exposure, adjusting for age, sex, estimated white blood cell proportions (3) and version of EPIC Chip used as fixed effect: CpG ~ age + sex + 11 deconvoluted WBC proportions + EPIC(v1/v2)

#### **TiKoCo**

##### Cohort description

Prospective population-based cohort study with 4203 participants individuals ≥14 years in June/July 2020 (at baseline), established to determine the SARS-CoV-2 seroprevalence in the county of Tirschenreuth, with two follow-up investigations four and nine months after baseline (12). All three time-points include questionnaire information and antibody measurements. For additional details refer to (13).

##### Variable definition and EWAS model

- Chronic disease: self-reported prevalent chronic disease (autoimmune, cancer, cardiovascular or diabetes).
- Smoking: self-reported
- Case definition: positive serology test.
- Control definition: negative serology results.
- Cases and controls were frequency-matched based on sex and age.
- Epigenome-wide analysis of methylation sites was conducted using linear models with DNAm as outcome and case status as exposure, adjusting for age, sex, estimated white blood cell proportions (3):

CpG ~ age + sex + 11 deconvoluted WBC proportions

## Supplemental Note 2: DNA methylation preprocessing and QC

Whole blood or DNA samples from the selected cohorts were shipped to HMGU, with details for each cohort described in the following subsections. Samples meeting basic quality measures were used for DNA-methylation (DNAm) analyses. For each sample from all included cohorts, 750 ng of genomic DNA was used for bisulfite conversion with the EZ-96 DNA Methylation Kit (Zymo Research, Orange, CA, USA). Subsequent methylation analysis was performed on an Illumina (San Diego, CA, USA) iScan platform using the Infinium MethylationEPIC BeadChip according to standard protocols provided by Illumina. GenomeStudio software version 2011.1 with Methylation Module version 1.9.0 was used for initial quality control of assay performance and for generation of methylation data export files.

- CONVINCE: Whole blood samples from 100 individuals were shipped to HMGU and genomic DNA extracted; genomic DNA from 100 samples was used as a starting point for the bisulfite conversion.
- LifeLines: Genomic DNA from 168 samples was shipped to HMGU and used as a starting point for the bisulfite conversion.
- NAKO: Buffy coat samples from 214 NAKO participants were processed to extract genomic DNA; aliquots of genomic DNA were available for 341 NAKO participants. Genomic DNA was used as a starting point for the bisulfite conversion.
- Tirschenreuther Study: Genomic DNA from 200 samples was shipped to HMGU and used as a starting point for the bisulfite conversion.

Further quality control and preprocessing of the data were performed in R v4.1.3 (R Core Team (2017). R: A language and environment for statistical computing. R Foundation for Statistical Computing, Vienna, Austria. URL https://www.R-project.org/), with the package minfi v1.40.0 (14) and following primarily the CPACOR pipeline (15). Raw intensities were read into R (command read.metharray) and background corrected (bgcorrect.illumina). Of note, the cut-off for the detection rate in each study is determined individually, considering the results from the sex prediction and median intensity quality control steps. The goal is to optimize the sample size while maintaining data quality, which is why the acceptable range for this threshold typically falls between 80% and 95%.

#### **CON-VINCE**

The detection rate threshold, considering the autosomes only, was set to 85% and all samples passed that threshold. A total of 60 186 probes were removed (some overlapping multiple categories): cross-reactive probes as given in published lists (N=44 493, (16, 17) ); probes with SNPs with minor allele frequency >5% at the CG position (N=11 370) or the single base extension (N=5597) as given by minfi; and 6228 with >5% missing values (autosomes only). A total of 805 673 probes remained for analysis.

No sample was removed prior to normalization, that is, all samples passed the sex prediction and median intensity quality control steps (implemented in minfi, commands getSex and getQC respectively), and had less than 15% missing values on the autosomes.

Quantile normalization (QN, R package limma v3.50.3 (18)) was then performed separately on the signal intensities divided into the 6 probe types: type II red, type II green, type I green unmethylated, type I green methylated, type I red unmethylated, type I red methylated (15). For the autosomes, QN was performed for all samples together; for the X and Y chromosomes, men and women were processed separately. The transformed intensities were then used to generate methylation beta values, a measure from 0 to 1 indicating the percentage of cells methylated at a given locus. Probes from the X chromosome (N=17743, following quality control) and the Y chromosome (N=379) are to be excluded from further analysis.

**Probe count summary:** The array contains 865 859 probes (846 232 on the autosomes, 19 090 on the X chromosome, 537 on the Y chromosome). 44 493 were cross-reactive probes, and 11 370 and 5597 had SNPs in the CG position and single base extensions respectively, and 6228 failed the detection p-value filter, a total of 62 477. However, many probes overlapped multiple categories: a total of 60 186 were removed. This leaves a total of 805 673 probes: 787 551 from the autosomes, 17743 from the X chromosome, 379 from the Y chromosome.

Notes on probe count: the original EPIC array had 866 895 probes, of which 59 are rs SNP probes for quality control. A “Product Quality Notice” (Tracking Number: PQN0223) issued by Illumina on April 19, 2017 indicated that 977 probes were removed due to underperformance, hence the total of 865 859.

**Sample count summary:** 100 samples were measured, and all passed quality control according to the cut-offs described above.

#### **LifeLines**

The detection rate threshold, considering the autosomes only, was set to 80% and 5 samples fell below that threshold. A total of 60 186 probes were removed (some overlapping multiple categories): cross-reactive probes as given in published lists (N=44 493, (16, 17) ); probes with SNPs with minor allele frequency >5% at the CG position (N=11370) or the single base extension (N=5597) as given by minfi; and 18283 with >10% missing values (autosomes only).

Five samples were removed prior to normalization: two for sex prediction failure and five for not passing median intensity quality control, as implemented in minfi (commands getSex and getQC respectively). The detection rate threshold (testing the autosomes only) was set to 80%, so that samples with greater than 20% missing values on the autosomes were removed (N=5, which all overlap with those failing the median intensity QC step).

Quantile normalization (QN, R package limma v3.50.3 (18)) was then performed separately on the signal intensities divided into the 6 probe types: type II red, type II green, type I green unmethylated, type I green methylated, type I red unmethylated, type I red methylated (15). For the autosomes, QN was performed for all samples together; for the X and Y chromosomes, men and women were processed separately. The transformed intensities were then used to generate methylation beta values, a measure from 0 to 1 indicating the percentage of cells methylated at a given locus. Probes from the X chromosome (N=17743, following quality control) and the Y chromosome (N=379) are to be excluded from further analysis.

**Probe count summary:** The array contains 865 859 probes (846 232 on the autosomes, 19 090 on the X chromosome, 537 on the Y chromosome). 44 493 were cross-reactive probes, and 11 370 and 5597 had SNPs in the CG position and single base extensions respectively, and 18 283 failed the detection p-value filter, a total of 79 743. However, many probes overlapped multiple categories: a total of 71 386 were removed. This leaves a total of 794 473 probes: 776 351 from the autosomes, 17 743 from the X chromosome, 379 from the Y chromosome.

Notes on probe count: the original EPIC array had 866 895 probes, of which 59 are rs SNP probes for quality control. A “Product Quality Notice” (Tracking Number: PQN0223) issued by Illumina on April 19, 2017 indicated that 977 probes were removed due to underperformance, hence the total of 865 859.

**Sample count summary:** 167 individuals were measured. Two were removed due to sex mismatch, 5 were removed due to failing quality control on the raw intensities and 5 failed the detection p-value filter (2 overlap with sex mismatch filter and complete overlap with intensity filter), leaving 162 individuals passing quality control.

#### **NAKO / NAPKON**

The detection rate threshold, considering the autosomes only, was set to 90% and 12 samples fell below that threshold. A total of 60 186 probes were removed (some overlapping multiple categories): cross-reactive probes as given in published lists (N=44 493, (16, 17) ); probes with SNPs with minor allele frequency >5% at the CG position (N=11 370) or the single base extension (N=5597) as given by minfi; and 22 602 with >15% missing values (autosomes only). A total of 789 299 probes remained for analysis.

12 samples were removed prior to normalization: four for sex prediction failure and 12 for not passing median intensity quality control, as implemented in minfi (commands getSex and getQC respectively). The detection rate threshold (testing the autosomes only) was set to 80%, so that samples with greater than 20% missing values on the autosomes were removed (N=12, which all overlap with those failing the median intensity QC step).

Quantile normalization (QN, R package limma v3.50.3 (18)) was then performed separately on the signal intensities divided into the 6 probe types: type II red, type II green, type I green unmethylated, type I green methylated, type I red unmethylated, type I red methylated (15). For the autosomes, QN was performed for all samples together; for the X and Y chromosomes, men and women were processed separately. The transformed intensities were then used to generate methylation beta values, a measure from 0 to 1 indicating the percentage of cells methylated at a given locus. Probes from the X chromosome (N=17743, following quality control) and the Y chromosome (N=379) are to be excluded from further analysis.

**Probe count summary:** The array contains 865 859 probes (846 232 on the autosomes, 19 090 on the X chromosome, 537 on the Y chromosome). 44 493 were cross-reactive probes, and 11 370 and 5597 had SNPs in the CG position and single base extensions respectively, and 18 283 failed the detection p-value filter, a total of 79 743. However, many probes overlapped multiple categories: a total of 87 760 were removed. This leaves a total of 789 299 probes.

Notes on probe count: the original EPIC array had 866 895 probes, of which 59 are rs SNP probes for quality control. A “Product Quality Notice” (Tracking Number: PQN0223) issued by Illumina on April 19, 2017 indicated that 977 probes were removed due to underperformance, hence the total of 865 859.

**Sample count summary:** 797 individuals were measured. Four were removed due to sex mismatch, 10 were removed due to failing quality control on the raw intensities and 10 failed the detection p-value filter (4 overlap with sex mismatch filter and complete overlap with intensity filter).

#### **Tirschenreuther Study**

The detection rate threshold, considering the autosomes only, was set to 95% and all samples passed that threshold.

A total of 61 824 probes were removed (some overlapping multiple categories): cross-reactive probes as given in published lists (N=44 493, (16, 17) ); probes with SNPs with minor allele frequency >5% at the CG position (N=11370) or the single base extension (N=5597) as given by minfi; and 8781 with >5% missing values (autosomes only).

No sample was removed prior to normalization, that is, all samples passed the sex prediction and median intensity quality control steps (implemented in minfi, commands getSex and getQC respectively), and had less than 5% missing values on the autosomes.

Quantile normalization (QN, R package limma v3.50.3 (18)) was then performed separately on the signal intensities divided into the 6 probe types: type II red, type II green, type I green unmethylated, type I green methylated, type I red unmethylated, type I red methylated (15). For the autosomes, QN was performed for all samples together; for the X and Y chromosomes, men and women were processed separately. The transformed intensities were then used to generate methylation beta values, a measure from 0 to 1 indicating the percentage of cells methylated at a given locus. Probes from the X chromosome (N=17743, following quality control) and the Y chromosome (N=379) are to be excluded from further analysis.

**Probe count summary:** The array contains 865 859 probes (846 232 on the autosomes, 19 090 on the X chromosome, 537 on the Y chromosome). 44 493 were cross-reactive probes, and 11 370 and 5 597 had SNPs in the CG position and single base extensions respectively, and 8781 failed the detection p-value filter, a total of 64 953. However, many probes overlapped multiple categories: a total of 61824 were removed. This leaves a total of 804 035 probes: 785913 from the autosomes, 17743 from the X chromosome, 379 from the Y chromosome.

Notes on probe count: the original EPIC array had 866 895 probes, of which 59 are rs SNP probes for quality control. A “Product Quality Notice” (Tracking Number: PQN0223) issued by Illumina on April 19, 2017 indicated that 977 probes were removed due to underperformance, hence the total of 865 859.

**Sample count summary:** 200 samples were measured, and all passed quality control according to the cut-offs described above.

## Supplemental Note 3: KORA FF4 DNA methylation and expression data

The Cooperative Health Research in the Region of Augsburg (KORA) FF4 (2013–2014) study is the second follow-up of the KORA S4 study (1999–2001) ([KORA cohort (Cooperative Health Research in the Region of Augsburg)](https://www.helmholtz-munich.de/en/epi/cohort/kora).

**FF4 DNA methylation data**

Genomic DNA (750 ng) from 1928 individuals was bisulfite converted using the EZ-96 DNA Methylation Kit (Zymo Research, Orange, CA, USA) in two separate batches (N=488, N=1440). Subsequent methylation analysis was performed on an Illumina (San Diego, CA, USA) iScan platform using the Infinium MethylationEPIC BeadChip according to standard protocols provided by Illumina. GenomeStudio software version 2011.1 with Methylation Module version 1.9.0 was used for initial quality control of assay performance and for generation of methylation data export files.

Further quality control and preprocessing of the data were performed in R v3.5.1 (R Core Team (2017). R: A language and environment for statistical computing. R Foundation for Statistical Computing, Vienna, Austria. URL https://www.R-project.org/), with the package minfi v1.28.3 (14) and following primarily the CPACOR pipeline (15). Raw intensities were read into R (command read.metharray) and background corrected (bgcorrect.illumina). Probes with detection p-values >0.01 were set to missing.

Before normalization, we removed problematic samples and probes. Forty samples were removed: 2 showed a mismatch between reported sex and that predicted by minfi; 33 had median intensity <50% of the experiment-wide mean, or <2000 arbitrary units; and 9 (overlap of 4 with previous) had >5% missing values on the autosomes. A total of 59631 probes were removed (some overlapping multiple categories): cross-reactive probes as given in published lists (N=44493, (16, 17)); probes with SNPs with minor allele frequency >5% at the CG position (N=11370) or the single base extension (N=5597) as given by minfi; and 5786 with >5% missing values. A total of 806228 probes remained for analysis.

Quantile normalization (QN) was then performed separately on the signal intensities divided into the 6 probe types: type II red, type II green, type I green unmethylated, type I green methylated, type I red unmethylated, type I red methylated (15). For the autosomes, QN was performed for all samples together; for the X and Y chromosomes, men and women were processed separately.

**FF4 gene expression data**

After RNA isolation using PAXgene Blood RNA Kit, RNA integrity number (RIN) was measured using the Agilent 2100 Bioanalyzer system. RNA samples with RIN values of approximately 6 or more were selected for mRNA sequencing (poly-A selected). The libraries were prepared using the Illumina stranded mRNA prep ligation kit (Illumina), following the kit's instructions. After a final QC, the libraries were sequenced in a paired-end mode (2x100 bases) in the Novaseq6000 sequencer (Illumina) with a depth of ≥ 40 Million reads per sample. After demultiplexing, FASTQ files from each sample are processed using standard tools. Alignment to UCSC Genome Browser hg19 human reference genome using STAR v2.4.2a (19). Unaligned reads are discarded. Sequencing QC was done using RNASeQC v1.1.8.1 (20). Properly aligned reads are then processed with HTSeq-count v0.6.1 (21) to generate read counts which can be interpreted as quantified gene expression. The reads are then normalized for exon length and total sequencing yield to generate Fragments Per Kilobase of transcript per Million mapped reads (FPKM), and this is done through dividing the fragments per gene by the product of length of the gene in kilobase and million reads sequenced.

After sequencing QC, samples QC was done. Samples with < 30 million reads were discarded. Exonic, intronic, intragenic, intergenic and rRNA rates calculated by RNAseQC were examined for outliers but no such outliers were found, and no samples were excluded based on these. Only the genes with FPKM of ≥ 1 in at least 5% of the samples were selected. Number of the selected genes in each sample were calculated. Samples having less than 5750 genes were excluded. Sex mismatches in the phenotype tables and those discerned from looking at the expression of XIST and UTY genes were also excluded.

# Group authorships

- **ORCHESTA working group:** Lifelines Corona Research Initiative, TiKoCo Working Group, CON-VINCE Consortium, NAKO Investigators
- **Lifelines Corona Research Initiative:** H. Marike Boezen^†1^, Jochen O. Mierau^2,3,9^, H. Lude Franke^4^, Jackie Dekens^4,6^, Patrick Deelen^4^, Pauline Lanting^4^, Judith M. Vonk^1^, Ilja Nolte^1^, Anil P.S. Ori^4,5^, Annique Claringbould^4^, Floranne Boulogne^4^, Marjolein X.L. Dijkema^4^, Henry H. Wiersma^4^, Robert Warmerdam^4^, Soesma A. Jankipersadsing^4^, Irene van Blokland^4,7^, Geertruida H. de Bock^1^, Judith GM Rosmalen^5,8^, Cisca Wijmenga^4^
  ^1^ Department of Epidemiology, University of Groningen, University Medical Center Groningen, Groningen, The Netherlands
  ^2^ Department of Economics, Econometrics & Finance, Faculty of Economics and Business, University of Groningen, Groningen, The Netherlands
  ^3^ Lifelines Cohort Study and Biobank, Groningen, The Netherlands
  ^4^ Department of Genetics, University of Groningen, University Medical Center Groningen, Groningen, The Netherlands
  ^5^ Department of Psychiatry, University of Groningen, University Medical Center Groningen, Groningen, The Netherlands
  ^6^ Center of Development and Innovation, University of Groningen, University Medical Center Groningen, Groningen, The Netherlands
  ^7^ Department of Cardiology, University of Groningen, University Medical Center Groningen, Groningen, The Netherlands
  ^8^ Department of Internal Medicine, University of Groningen, University Medical Center Groningen, Groningen, The Netherlands
  ^9^ Team Strategy &External Relations, University of Groningen, University Medical Center Groningen, the Netherlands
- **TiKoCo Working Group:**

Analysis team lead: Iris M. Heid^1^; Data collection lead: Ralf Wagner^2,3^ & Klaus Überla^4^; Analysis team member Thomas W. Winkler^1^, Simon Wiegrebe^1,5^, Mathias Gorski^1^ & Klaus J. Stark^1^; Data collection member: David Peterhoff^2,3^, Sebastian Einhauser^2^, Hans-Helmut Niller^2^ & Stephanie Beileke^4^

^1^Department of Genetic Epidemiology, University of Regensburg, Regensburg, Germany

^2^Institute of Medical Microbiology and Hygiene, Molecular Microbiology (Virology), University of Regensburg, Regensburg, Germany

^3^Institute of Clinical Microbiology and Hygiene, University Hospital Regensburg, Regensburg, Germany

^4^Institute of Clinical and Molecular Virology, University Hospital Erlangen, Friedrich-Alexander-Universität Erlangen-Nürnberg, Erlangen, Germany

^5^Statistical Consulting Unit StaBLab, Department of Statistics, Ludwig-Maximilians-Universität (LMU) Munich, Munich, Germany

- **CON-VINCE Consortium**:

Tamir Abdelrahman^1^, Geeta Acharya^2^, Gloria Aguayo^2^, Pinar Alper^3^, Wim Ammerlaan^2^, François Ancien^3^, Ariane Assele-Kama^2^, Christelle Bahlawane^2^, Katy Beaumont^2^, Nadia Beaupain^2^, Lucrèce Beckers^2^, Camille Bellora^2^, Fay Betsou^2^, Luc Biver^4^, Sandie Boly^2^, Dirk Brenner^2^, Henry-Michel Cauchie^5^, Eleftheria Charalambous^2^, Emilie Charpentier^2^, Estelle Coibion^1^, Sylvie Coito^6^, Delphine Collart^5^, Manuel Counson^2^, Brian De Witt^2^, Antonelle Di Pasquale^4^, Olivia Domingues^2^, Claire Dording^2^, Jean-Luc Dourson^7^, Bianca Dragomir^2^, Tessy Fautsch^2^, Jean-Yves Ferrand^2^, Thibault Ferrandon^7^, Ana Festas Lopes^2^, Guillaume Fournier^1^, Joëlle Véronique Fritz^2^, Manon Gantenbein^2^, Piotr Gawron^3^, Laura Georges^2^, Soumyabrata Ghosh^3^, Stéphane Gidenne^6^, Enrico Glaab^3^, Clarissa P. C. Gomes^3^, Borja Gomez Ramos^3^, Vyron Gorgogietas^3^, Jérôme Graas^2^, Valentin Groues^3^, Wei Gu^3^, Gael Hamot^2^, Anne-Marie Hanff^2^, Maxime Hansen^2^, Linda Hansen^8^, Lisa Hefele^2^, Laurent Heirendt^3^, Ahmed Hemedan^3^, Estelle Henry^2^, Margaux Henry^2^, Eve Herkenne^2^, Sascha Herzinger^3^, Christiane Hilger^2^, Laetitia Huiart^2^, Alexander Hundt^2^, Judith Hübschen^2^, Gilles Iserentant^2^, Philipp Jägi^9^, Anne Kaysen^3^, Piyapong Khurmin^3^, Fédéric Klein^7^, Tommy Klein^4^, Stéphanie Kler^2^, Alexey Kolodkin^2^, Rejko Krüger^2^, Pauline Lambert^2^, Jacek Jaroslaw Lebioda^3^, Sabine Lehmann^2^, Marie Leick^1^, Anja Leist^10^, Morgane Lemaire^2^, Andrew Lumley^2^, Annika Lutz^10^, João Manuel Loureiro^8^, Monica Marchese^2^, Tainà Marques^3^, François Massart^3^, Patrick May^3^, Maura Minelli^2^, Alessandra Mousel^2^, Maeva Munsch^2^, Sophie Mériaux^2^, Friedrich Mühlschlegel^1^, Mareike Neumann^2^, Trang Nguyen^2^, Beatrice Nicolai^8^, Marc Paul O'Sullivan^2^, Leslie Ogorzaly^5^, Jochen Ohnmacht^2^, Christiane Olesky^3^, Markus Ollert^2^, Claire Pauly^3^, Laure Pauly^3^, Lukas Pavelka^3^, Christian Penny^5^, Magali Perquin^2^, Achilleas Pexaras^2^, Palma di Pinto^6^, Marie France Pirard^1^, Jean-Marc Plesseria^2^, Guilherme Ramos Meyers^3^, Armin Rauschenberger^3^, Lucie Remark^2^, Antonio Rodriguez^4^, Basile Rommes^3^, Kirsten Rump^3^, Estelle Sandt^2^, Bruno Santos^2^, Venkata P. Satagopam^3^, Aurélie Sausy^2^, Margaux Schmitt^2^, Christiane Schmitt^9^, Reinhard Schneider^3^, Valerie Schröder^3^, Serge Schumacher^4^, Alexandra Schweicher^8^, Sneeha Seal^2^, Jean-Yves Servais^2^, Florian Simon^2^, Amna Skrozic^3^, Chantal Snoeck^2^, Kate Sokolowska^2^, Lara Stute^3^, Hermann Thien^2^, Stéphane Toll^9^, Noua Toukourou^3^, Christophe Trefois^3^, Johanna Trouet^2^, Nguyen Trung^1^, Jonathan Turner^2^, Michel Vaillant^2^, Daniela Valoura Esteves^2^, Carlos Vega Moreno^3^, Charlène Verschueren^2^, Maharshi Vyas^3^, Claus Vögele^10^, Cécile Walczak^5^, Xinhui Wang^3^, Femke Wauters^8^, Bernard Weber^9^, Emilie Weibel^7^, Tania Zamboni^2^

^1^Laboratoire National de Santé, Dudelange, Luxembourg

^2^Luxembourg Institute of Health, Strassen, Luxembourg

^3^Luxembourg Centre for Systems Biomedicine, University of Luxembourg, Esch-Belval, Luxembourg

^4^TNS-ILRES, Bertrange, Luxembourg

^5^Luxembourg Institute of Science and Technology, Luxembourg, Luxembourg

^6^Ketterthill, Belvaux, Luxembourg

^7^BioNeXt, Leudelange, Luxembourg

^8^Centre Hospitalier de Luxembourg, Luxembourg, Luxembourg

^9^Laboratoire Réunis, Junglinster, Luxembourg

^10^University of Luxembourg, Esch-sur-Alzette, Luxembourg

- **NAKO Investigators:**

Annette Peters^1,2^, Thomas Keil^3,4^, Lilian Krist^3^, Tobias Pischon^5,6^, Jean-Paul Hirschel^5^, Matthias Schulze^7,8^, Sylvia Gastell^9^, Wolfgang Ahrens^10,11^, Kathrin Günther^10^, Oliver Kuß^12,13^, Nina Ebert^12^, Tamara Schikowski^14^, Claudia Wigmann^14^, Börge Schmidt^15^, Andreas Stang^15^, Anna Köttgen^16^, Jasmin Kiekert^17^, Rafael Mikolajczyk^18^, Cornelia Gottschick^18^, Volker Harth^19^, Nadia Obi^20,19^, Stefanie Castell^21^, Carolina Klett-Tammen^21^, Wolfgang Lieb^22^, Cara Övermöhle^22^, Markus Löffler^23^, Kerstin Wirkner^24^, Rudolf Kaaks^25^, Verena Katzke^25^, Till Bärnighausen^26^, Heiko Becher^26^, André Karch^27^, Klaus Berger^27^, Henry Völzke^28,29^, Sabine Schipf^28^, Michael Leitzmann^30^, Beate Fischer^30^, Hermann Brenner^31^, Lena Koch-Gallenkamp^31^, Bernd Holleczek^32^

^1^Institute of Epidemiology, Helmholtz Zentrum München - German Research Center for Environmental Health (GmbH), Neuherberg, Germany

^2^Chair of Epidemiology, Institute for Medical Information Processing, Biometry and Epidemiology, Ludwig-Maximilians-Universität München, Munich, Germany

^3^Institute of Social Medicine, Epidemiology and Health Economics, Charité – Universitätsmedizin Berlin, Berlin, Germany

^4^Institute of Clinical Epidemiology and Biometry, University of Wuerzburg, Würzburg, Germany

^5^Max Delbrück Center for Molecular Medicine in the Helmholtz Association (MDC), Molecular Epidemiology Research Group, Berlin, Germany

^6^Max Delbrück Center for Molecular Medicine in the Helmholtz Association (MDC), Biobank Technology Platform, Berlin, Germany

^7^Department of Molecular Epidemiology, German Institute of Human Nutrition Potsdam-Rehbruecke (DIfE), Nuthetal, Germany

^8^Institute of Nutritional Science, University of Potsdam

^9^NAKO Study Center, German Institute of Human Nutrition Potsdam-Rehbruecke, Nuthetal, Germany

^10^Leibniz Institute for Prevention Research and Epidemiology – BIPS, Nuthetal, Germany, ^11^University Bremen, Bremen, Germany

^12^Institute for Biometrics and Epidemiology, German Diabetes Center, Leibniz Center for Diabetes Research at Heinrich Heine University, Düsseldorf, Germany

^13^Centre for Health and Society, Medical Faculty and University Hospital Düsseldorf, Heinrich Heine University Düsseldorf, Düsseldorf, Germany

^14^IUF - Leibniz Research Institute for Environmental Medicine, Düsseldorf, Germany

^15^Institute for Medical Informatics, Biometry and Epidemiology, University Hospital Essen, University of Duisburg-Essen, Essen, Germany

^16^Institute of Genetic Epidemiology, Faculty of Medicine and Medical Center, University of Freiburg, Freiburg, Germany

^17^Institute for Prevention and Cancer Epidemiology, Faculty of Medicine and Medical Center, University of Freiburg, Freiburg, Germany

^18^Institute of Medical Epidemiology, Biometrics and Informatics, Medical Faculty of the Martin-Luther University Halle-Wittenberg, Halle (Saale), Germany

^19^Institute for Occupational and Maritime Medicine Hamburg (ZfAM), University Medical Centre Hamburg-Eppendorf (UKE), Hamburg, Germany

^20^Institute for Medical Biometry and Epidemiology, University Medical Center Hamburg-Eppendorf, Hamburg, Germany

^21^Department for Epidemiology, Helmholtz Centre for Infection Research (HZI), Greifswald, Germany

^22^Institute of Epidemiology, Kiel University, Kiel, Germany

^23^Institute for Medical Informatics, Statistics and Epidemiology, University of Leipzig, Leipzig, Germany

^24^University of Leipzig, Medical Faculty, Leipzig, Germany

^25^Division of Cancer Epidemiology, German Cancer Research Center (DKFZ), Heidelberg, Germany

^26^Heidelberg Institute of GlobalHealth, University Hospital Heidelberg, Heidelberg, Germany

^27^Institute of Epidemiology and Social Medicine, University of Münster, Münster, Germany

^28^Institute for Community Medicine, University Medicine Greifswald, Greifswald, Germany

^29^German Centre for Cardiovascular Research (DZHK), ^30^Institute for Epidemiology and Preventive Medicine, University of Regensburg, Regensburg, Germany

^31^Division of Clinical Epidemiology and Aging Research, German Cancer Research Center (DKFZ), Division of Preventive Oncology, German Cancer Research Center (DKFZ), Heidelberg, Germany

^32^Saarland Cancer Registry, Saarbrücken, Germany

# References

1. Elizarraras JM, Liao Y, Shi Z, Zhu Q, Pico Alexander R, Zhang B. WebGestalt 2024: faster gene set analysis and new support for metabolomics and multi-omics. Nucleic Acids Research. 2024;52(W1):W415-W21.

2. Tsurkalenko O, Bulaev D, O’Sullivan MP, Snoeck C, Ghosh S, Kolodkin A, et al. Creation of a pandemic memory by tracing COVID-19 infections and immunity in Luxembourg (CON-VINCE). BMC Infectious Diseases. 2024;24(1):179.

3. Salas LA, Zhang Z, Koestler DC, Butler RA, Hansen HM, Molinaro AM, et al. Enhanced cell deconvolution of peripheral blood using DNA methylation for high-resolution immune profiling. Nat Commun. 2022;13(1):761.

4. Stolk RP, Rosmalen JGM, Postma DS, de Boer RA, Navis G, Slaets JPJ, et al. Universal risk factors for multifactorial diseases. European Journal of Epidemiology. 2008;23(1):67-74.

5. Sijtsma A, Rienks J, van der Harst P, Navis G, Rosmalen JGM, Dotinga A. Cohort Profile Update: Lifelines, a three-generation cohort study and biobank. International journal of epidemiology. 2021;51(5):e295-e302.

6. Scholtens S, Smidt N, Swertz MA, Bakker SJ, Dotinga A, Vonk JM, et al. Cohort Profile: LifeLines, a three-generation cohort study and biobank. International journal of epidemiology. 2015;44(4):1172-80.

7. Mc Intyre K, Lanting P, Deelen P, Wiersma HH, Vonk JM, Ori APS, et al. Lifelines COVID-19 cohort: investigating COVID-19 infection and its health and societal impacts in a Dutch population-based cohort. BMJ Open. 2021;11(3):e044474.

8. Peters A, Peters A, Greiser KH, Göttlicher S, Ahrens W, Albrecht M, et al. Framework and baseline examination of the German National Cohort (NAKO). Eur J Epidemiol. 2022;37(10):1107-24.

9. German National Cohort C. The German National Cohort: aims, study design and organization. European Journal of Epidemiology. 2014;29(5):371-82.

10. Mikolajczyk R, Diexer S, Fricke J, Ahnert P, Pischon T, Karch A. Description of the COVID 2.0 survey in the NAKO and first results. European Journal of Public Health. 2023;33(Supplement_2).

11. Mikolajczyk R, Diexer S, Klee B, Pfrommer L, Purschke O, Fricke J, et al. Likelihood of Post-COVID Condition in people with hybrid immunity; data from the German National Cohort (NAKO). Journal of Infection. 2024;89(2).

12. Wagner R, Peterhoff D, Beileke S, Günther F, Berr M, Einhauser S, et al. Estimates and Determinants of SARS-Cov-2 Seroprevalence and Infection Fatality Ratio Using Latent Class Analysis: The Population-Based Tirschenreuth Study in the Hardest-Hit German County in Spring 2020. Viruses. 2021;13(6).

13. Einhauser S, Peterhoff D, Beileke S, Günther F, Niller HH, Steininger P, et al. Time Trend in SARS-CoV-2 Seropositivity, Surveillance Detection- and Infection Fatality Ratio until Spring 2021 in the Tirschenreuth County-Results from a Population-Based Longitudinal Study in Germany. Viruses. 2022;14(6).

14. Aryee MJ, Jaffe AE, Corrada-Bravo H, Ladd-Acosta C, Feinberg AP, Hansen KD, et al. Minfi: a flexible and comprehensive Bioconductor package for the analysis of Infinium DNA methylation microarrays. Bioinformatics. 2014;30(10):1363-9.

15. Lehne B, Drong AW, Loh M, Zhang W, Scott WR, Tan ST, et al. A coherent approach for analysis of the Illumina HumanMethylation450 BeadChip improves data quality and performance in epigenome-wide association studies. Genome Biol. 2015;16(1):37.

16. Pidsley R, Zotenko E, Peters TJ, Lawrence MG, Risbridger GP, Molloy P, et al. Critical evaluation of the Illumina MethylationEPIC BeadChip microarray for whole-genome DNA methylation profiling. Genome Biol. 2016;17(1):208.

17. McCartney DL, Walker RM, Morris SW, McIntosh AM, Porteous DJ, Evans KL. Identification of polymorphic and off-target probe binding sites on the Illumina Infinium MethylationEPIC BeadChip. Genom Data. 2016;9:22-4.

18. Ritchie ME, Phipson B, Wu D, Hu Y, Law CW, Shi W, et al. limma powers differential expression analyses for RNA-sequencing and microarray studies. Nucleic Acids Res. 2015;43(7):e47.

19. Dobin A, Davis CA, Schlesinger F, Drenkow J, Zaleski C, Jha S, Batut P, Chaisson M, Gingeras TR. STAR: ultrafast universal RNA-seq aligner.Bioinformatics. 2013 Jan 1;29(1):15-21.

20. DeLuca DS, Levin JZ, Sivachenko A, Fennell T, Nazaire MD, Williams C, Reich M, Winckler W, Getz G. RNA-SeQC: RNA-seq metrics for quality control and process optimization. Bioinformatics. 2012 Jun 1;28(11):1530-2.

21. Anders S, Pyl PT, Huber W. HTSeq--a Python framework to work with high-throughput sequencing data.Bioinformatics. 2015 Jan 15;31(2):166-9.
